# Precision Medicine in Non-Hodgkin Lymphoma: Advances in BTK Inhibition, CD30-Directed Antibody–Drug Conjugates, and HDAC-Mediated Epigenetic Therapy with Pirtobrutinib, Brentuximab Vedotin, and Belinostat

**DOI:** 10.3390/jcm15124425

**Published:** 2026-06-08

**Authors:** Piotr Kawczak, Tomasz Bączek

**Affiliations:** 1 Department of Pharmaceutical Chemistry, Faculty of Pharmacy, Medical University of Gdańsk, 80-416 Gdańsk, Poland; tomasz.baczek@gumed.edu.pl; 2 Department of Nursing and Medical Rescue, Institute of Health Sciences, Pomeranian University in Słupsk, 76-200 Słupsk, Poland

**Keywords:** non-Hodgkin lymphoma, pirtobrutinib, Bruton tyrosine kinase inhibitors, brentuximab vedotin, antibody–drug conjugates, belinostat, histone deacetylase inhibitors, targeted therapy, personalized oncology, molecular therapeutics, precision medicine

## Abstract

Non-Hodgkin lymphoma (NHL) encompasses a biologically diverse group of malignancies for which the integration of precision medicine has markedly reshaped therapeutic strategies. Recent advances in molecular profiling, target identification, and drug development have led to the introduction of highly selective agents capable of overcoming resistance mechanisms and improving outcomes in relapsed or refractory disease. This review highlights three targeted therapies—pirtobrutinib, brentuximab vedotin, and belinostat—and their evolving roles in modern NHL management. Pirtobrutinib, a next-generation, non-covalent Bruton tyrosine kinase (BTK) inhibitor, demonstrates preserved activity in patients previously treated with covalent BTK inhibitors (BTKi), addressing a critical unmet need in B-cell lymphomas. Brentuximab vedotin, an antibody–drug conjugate targeting CD30, has significantly improved therapeutic precision by delivering cytotoxic agents directly to lymphoma cells and has become a central component of treatment for CD30-expressing NHL subtypes. Belinostat, a broad-spectrum histone deacetylase (HDAC) inhibitor, offers a mechanistically distinct epigenetic approach, particularly in peripheral T-cell lymphomas (PTCL), where conventional chemotherapy has limited efficacy. Together, these agents exemplify three complementary paradigms of precision oncology in NHL: kinase signaling inhibition, antigen-directed cytotoxic delivery, and epigenetic modulation. This review synthesizes current evidence, clinical trial data, and future perspectives regarding the integration of pirtobrutinib, brentuximab vedotin, and belinostat into evolving treatment paradigms. Cumulatively, these therapies illustrate both the progress and the ongoing challenges of biomarker-driven treatment in NHL, including resistance mechanisms, toxicity management, optimal therapeutic sequencing, and variability in evidence maturity across targeted strategies. While pirtobrutinib and brentuximab vedotin are supported by increasingly robust clinical evidence in selected lymphoma subtypes, the role of belinostat remains constrained by modest response rates and limited randomized data, underscoring the continued need for biomarker refinement and more precisely individualized therapeutic approaches in NHL precision medicine.

## 1. Introduction

NHL comprises a biologically and clinically heterogeneous group of lymphoid malignancies arising from B-cell, T-cell, or natural killer (NK)-cell lineages and represents one of the most common hematologic cancers worldwide [1,2,3]. This diversity reflects the complexity of lymphocyte ontogeny, encompassing distinct differentiation states, genomic alterations, and microenvironmental dependencies that, in combination, shape disease behavior and therapeutic responsiveness. Advances in modern classification systems integrating molecular, genetic, and immunophenotypic features have substantially refined diagnostic precision and clarified the prognostic and therapeutic relevance of individual subtypes [3,4,5], with ongoing efforts to further bridge biological insights and disease taxonomy [6]. In parallel, contemporary clinical practice has evolved toward subtype-specific, guideline-driven management frameworks, as reflected in recommendations from organizations such as the National Comprehensive Cancer Network (NCCN) and European Society for Medical Oncology (ESMO) [1,2]. As a result, NHL is increasingly conceptualized not as a single disease entity but as a spectrum of biologically defined malignancies characterized by distinct molecular vulnerabilities and therapeutic targets [7,8,9], supported by expanding therapeutic platforms including immunotherapy, antibody-based strategies, and cellular therapies [10,11].

Despite these advances, conventional cytotoxic chemotherapy—most notably anthracycline-based regimens such as CHOP and rituximab-containing R-CHOP—has historically constituted the backbone of treatment for many aggressive B-cell lymphomas [12,13]. While curative outcomes are achievable in a proportion of patients, particularly in diffuse large B-cell lymphoma (DLBCL), relapse and refractory disease remain major clinical challenges [13,14,15,16]. These limitations are driven in part by underlying biological heterogeneity, clonal evolution under treatment pressure, and the emergence of resistant subclones, which together constrain the durability of non-specific cytotoxic approaches [5,15,17]. Moreover, clinically relevant molecular subtypes—including activated B-cell-like (ABC), germinal center B-cell-like (GCB), and double-hit lymphomas—exhibit distinct therapeutic sensitivities and prognoses, further underscoring the need for biologically informed treatment strategies [18,19,20,21,22]. Contemporary treatment paradigms increasingly incorporate targeted agents and novel combinations, aiming to improve outcomes beyond conventional chemotherapy backbones [23,24].

The increasing availability of high-throughput molecular technologies, including next-generation sequencing (NGS), transcriptomic profiling, and epigenetic analyses, has enabled deeper characterization of lymphoma biology and the identification of actionable therapeutic targets [25], with growing global implementation even in resource-variable settings [26]. Among these, dysregulation of B-cell receptor (BCR) signaling has emerged as a central pathogenic mechanism in many B-cell malignancies, promoting tumor survival, proliferation, and interaction with the tumor microenvironment [27,28]. Targeting this pathway has led to the development of Bruton’s tyrosine kinase (BTK) inhibitors, which have become integral components of modern lymphoma therapy [29,30,31]. However, resistance to first-generation covalent BTKi frequently arises, most commonly through mutations affecting the BTK C481 binding site, alongside alternative signaling adaptations [27,28,30]. These challenges have catalyzed the development of next-generation agents such as pirtobrutinib, a highly selective non-covalent BTK inhibitor capable of overcoming C481-mediated resistance and demonstrating clinically meaningful activity in heavily pretreated populations [32,33,34].

Complementing kinase inhibition, antibody–drug conjugates (ADCs) represent a distinct therapeutic strategy that couples antigen specificity with targeted cytotoxic delivery. Brentuximab vedotin, an anti-CD30 ADC linked to the microtubule-disrupting agent monomethyl auristatin E (MMAE), exemplifies this approach and has demonstrated significant efficacy in CD30-expressing lymphomas [35,36]. Although initially developed in Hodgkin lymphoma and systemic anaplastic large-cell lymphoma, its application has expanded to selected NHL subtypes, supported by both biological rationale and randomized clinical evidence in CD30-positive PTCL and emerging evidence in other NHL settings [35,36]. More broadly, ADCs and related antibody-based platforms—including bispecific T-cell engagers—are reshaping therapeutic algorithms across lymphoma subtypes [11,37]. Nevertheless, variability in antigen expression and toxicity profiles continues to influence optimal patient selection and clinical use.

In parallel, epigenetic dysregulation has emerged as a critical driver of lymphomagenesis, particularly in PTCL, where outcomes remain poor with conventional therapies [2,38]. Aberrant histone modification and chromatin remodeling contribute to oncogenic transcriptional programs and resistance mechanisms, providing a strong rationale for epigenetic-targeted therapies. HDAC inhibitors such as belinostat have demonstrated clinical activity in relapsed or refractory PTCL, although response rates remain modest and are supported primarily by non-randomized data, highlighting the need for improved patient selection and rational combination strategies [38,39,40]. Ongoing research is focused on integrating epigenetic agents with immunotherapy and targeted therapies to enhance therapeutic efficacy [41,42].

Beyond tumor-intrinsic alterations, the tumor microenvironment plays a pivotal role in disease progression, immune evasion, and therapeutic resistance through complex interactions between malignant cells and surrounding stromal and immune components [20,33,34]. These insights have contributed to the development of immunotherapeutic approaches, including immune checkpoint inhibition and cellular therapies, which have transformed outcomes in selected patient populations [20,43,44]. Advances in imaging and response assessment, particularly molecular imaging techniques, further support precision management and treatment monitoring [45]. Additionally, circulating tumor DNA (ctDNA) has emerged as a promising tool for non-invasive disease monitoring and dynamic assessment of treatment response, although its routine clinical application remains under active investigation [46,47].

Together, these developments reflect a paradigm shift from empiric, chemotherapy-based treatment toward precision medicine approaches that integrate molecular biology, disease subtype, and patient-specific factors into therapeutic decision-making [19,20,25]. However, important challenges persist, including the identification of robust predictive biomarkers, the management of primary and acquired resistance, optimal sequencing and combination of therapies, and disparities in access to advanced diagnostics and targeted agents across healthcare systems, with economic considerations increasingly influencing real-world implementation [7,8,9,48].

Against this evolving backdrop, pirtobrutinib, brentuximab vedotin, and belinostat were selected because they represent three mechanistically distinct yet complementary paradigms of precision medicine in NHL: targeted kinase signaling inhibition, antigen-directed antibody–drug conjugation, and epigenetic modulation. Although these agents differ substantially in disease context, biomarker dependence, and level of supporting clinical evidence, together they illustrate the heterogeneity of biomarker-driven therapeutic development across NHL subtypes and the differing degrees of clinical maturity among contemporary targeted strategies. Pirtobrutinib reflects the evolution of rationally designed next-generation kinase inhibitors developed to overcome acquired resistance mechanisms; brentuximab vedotin exemplifies the successful integration of biomarker-directed ADC therapy into randomized treatment frameworks in selected CD30-positive lymphomas; and belinostat highlights both the promise and the current limitations of epigenetic therapies supported primarily by single-arm clinical evidence. Together, these agents provide a focused lens through which to examine both the progress and the remaining challenges of precision oncology in NHL.

This narrative review aims to deliver a comprehensive and critically integrated overview of precision medicine in NHL, emphasizing the clinical and biological rationale underpinning these therapeutic strategies. By synthesizing mechanistic insights, pivotal clinical trial evidence, and real-world considerations, this work contextualizes the evolving role of pirtobrutinib, brentuximab vedotin, and belinostat within contemporary, guideline-informed treatment frameworks, while highlighting ongoing challenges and future opportunities in the optimization of personalized care for patients with lymphoid malignancies.

Relevant literature was identified through selective searches of the PubMed and Scopus databases focusing on English-language publications published between 2006 and April 2026. The reviewed literature included pivotal clinical trials, randomized studies, registration-supporting investigations, meta-analyses, major review articles, and clinically informative real-world studies relevant to pirtobrutinib, brentuximab vedotin, belinostat, and precision medicine approaches in NHL. Additional references were incorporated based on their clinical relevance, translational significance, or contribution to the contextual interpretation of evolving therapeutic strategies, resistance mechanisms, biomarker development, and treatment sequencing in lymphoma.

The schematic figures presented in this manuscript were developed by the authors using graphical design software that may include artificial intelligence–supported visualization tools. All scientific concepts, data interpretation, and final graphical compositions were independently evaluated, revised, and approved by the authors, who assume full responsibility for the accuracy, integrity, and originality of the presented material.

**Figure 1:**
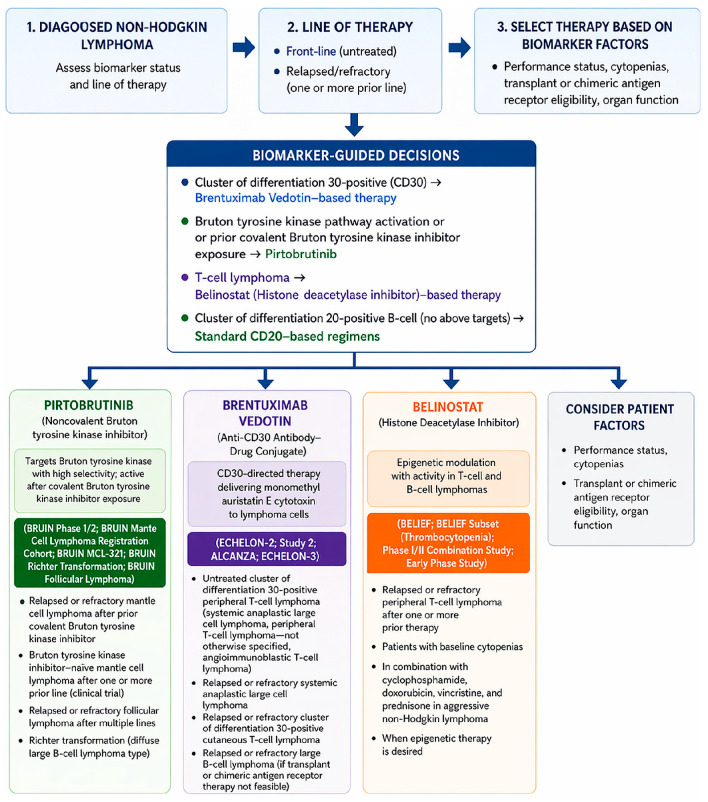
Schematic comparison overview of treatment selection in non-Hodgkin lymphoma, integrating pivotal clinical trial data by biomarker status and line of therapy with the use of pirtobrutinib, brentuximab vedotin, and belinostat. The schematic diagram represents an original, author-developed interpretative synthesis of currently available evidence in accordance with major NCCN and ESMO therapeutic principles, including biomarker-guided treatment stratification and sequential therapy planning. The figure constitutes an independent graphical conceptualization created for this manuscript and does not reproduce, duplicate, or directly adapt any previously published guideline, illustration, or source material. All abbreviations employed are defined in the text in the Abbreviations Section.

Figure 1 provides a schematic overview of treatment selection in NHL, integrating key clinical trial evidence according to biomarker status, disease subtype, and line of therapy.

## 2. Pirtobrutinib

Pirtobrutinib (LOXO-305) is a highly selective, next-generation, non-covalent (reversible) inhibitor of BTK, developed to overcome key limitations of first- and second-generation covalent BTKi such as ibrutinib and acalabrutinib [49,50]. Unlike covalent inhibitors that irreversibly bind the C481 residue of BTK, pirtobrutinib maintains activity independently of this binding site, thereby overcoming one of the most common mechanisms of acquired resistance—namely C481 mutations [51,52,53]. This pharmacologic distinction is clinically relevant in heavily pretreated populations with relapsed or refractory NHL and chronic lymphocytic leukemia (CLL), where resistance to covalent BTKi frequently limits long-term disease control [54,55]. Mechanistically, resistance to BTK inhibition reflects both on-target mutations and adaptive pathway reprogramming, underscoring the need for next-generation inhibitors such as pirtobrutinib [56,57].

**Figure 2:**
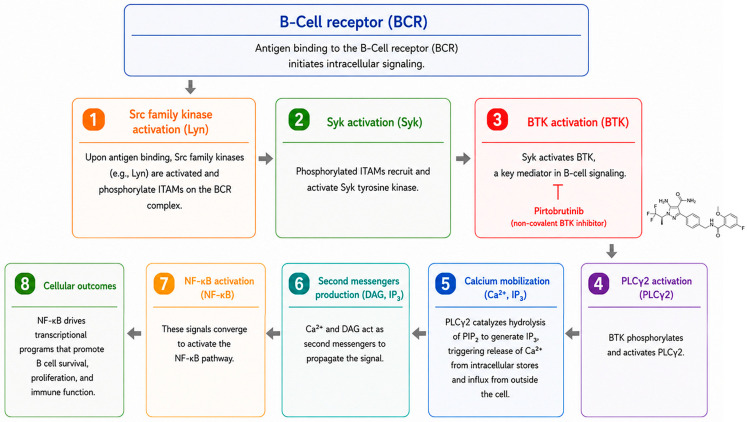
Pirtobrutinib mechanism of action. Original schematic illustration created by the authors based on the mechanism described in [68]. Pirtobrutinib is a next-generation, highly selective, non-covalent BTK inhibitor that effectively targets both wild-type and resistance-associated BTK mutations (including C481 variants—C481S, C481T, C481R), demonstrating potent antitumor activity with minimal off-target effects and the potential to overcome limitations of covalent BTK inhibitors. All abbreviations employed are defined in the text in the Abbreviations Section.

BTK is a central component of BCR signaling, regulating downstream pathways including NF-κB, PI3K/AKT, and MAPK, which in combination drive malignant B-cell proliferation, survival, and microenvironmental interactions [58,59,60]. Pirtobrutinib exerts potent and selective inhibition of BTK activity, demonstrating comparable efficacy against both wild-type and mutant BTK in preclinical models [61,62,63]. Its improved kinome selectivity relative to earlier BTKi reduces off-target effects, likely contributing to the favorable safety and tolerability profile observed in clinical studies [64], a feature emphasized in early clinical evaluations and regulatory approval analyses [65,66,67]. Figure 2 illustrates the mechanism of action of pirtobrutinib.

The first clinical evidence supporting pirtobrutinib was generated in the phase I/II BRUIN trial (NCT03740529), a multicenter, open-label, dose-escalation and expansion study evaluating its activity across multiple relapsed or refractory B-cell malignancies [32,69]. This study enrolled heavily pretreated patients, many of whom had prior exposure to covalent BTKi and other targeted therapies, with eligibility generally requiring at least one prior line of systemic treatment and, for the mantle cell lymphoma (MCL) cohort, prior covalent BTKi exposure. Notably, no maximum tolerated dose was identified, and the recommended phase II dose of 200 mg once daily was selected based on pharmacokinetic and pharmacodynamic considerations rather than dose-limiting toxicity [70,71], highlighting a potentially favorable pharmacologic profile relative to earlier BTKi.

Within BRUIN, pirtobrutinib demonstrated clinically meaningful activity across NHL subtypes. In MCL, ORRs of approximately 50–52% were observed in patients previously treated with covalent BTKi, with durable responses reported in heavily pretreated populations [72]. These findings were further supported in the MCL registration cohort (n ≈ 120), which reported an ORR of 50%, CR rate of 13%, and median duration of response (DoR) of 8.3 months, forming the basis for regulatory approval in this setting. Real-world and expanded-access data further support these observations, demonstrating activity in routine clinical practice [73]. Importantly, this cohort consisted largely of patients refractory to their last BTK inhibitor, underscoring the potential role of pirtobrutinib in a high-risk population characterized by limited therapeutic options. However, interpretation of these findings should recognize the single-arm design and absence of randomized comparator data.

Additional subgroup analyses from BRUIN extended these findings across disease contexts. In follicular lymphoma (FL), ORRs of approximately 50–52% (CR ~16.7%) with a median DoR of ~10.2 months were reported in patients with a median of three prior lines of therapy [74]. Similarly, in marginal zone lymphoma (MZL), response rates approached 50–55% with encouraging durability [75]. In the Richter transformation (RT) subgroup—an aggressive and biologically distinct entity—pirtobrutinib demonstrated antitumor activity and manageable tolerability despite the historically poor prognosis associated with this condition [76,77]. While these data suggest broad activity across both indolent and aggressive lymphoma subtypes, interpretation should be tempered by the single-arm design, limited sample sizes in certain cohorts, and potential for selection bias inherent to early-phase studies. Compared with antibody–drug conjugates or cellular immunotherapies, pirtobrutinib offers the practical advantage of oral administration and relatively predictable toxicity management, although response durability may remain more limited in biologically aggressive disease settings.

In parallel, the phase III BRUIN MCL-321 trial (NCT04662255) is evaluating pirtobrutinib versus investigator’s choice of covalent BTK inhibitor in previously treated, BTKi-naïve MCL, with progression-free survival (PFS) as the primary endpoint [78]. This global, randomized study specifically addresses an earlier-line population not exposed to prior BTK inhibition and is expected to provide confirmatory evidence while helping define the positioning of pirtobrutinib relative to first-generation BTKi. Comparative analyses with other advanced therapies, including CAR-T approaches, further highlight the need to contextualize pirtobrutinib within an increasingly competitive therapeutic landscape [79]. In contrast to CAR-T therapy, which may achieve deeper and potentially more durable remissions in selected patients, pirtobrutinib provides a more accessible and less logistically intensive treatment option, particularly for older or medically frail patients who may not be candidates for cellular therapy.

In CLL/SLL, pirtobrutinib has demonstrated ORRs exceeding 80% in patients previously exposed to covalent BTKi, with durable responses and favorable PFS outcomes [80,81]. These findings were corroborated in the randomized phase III BRUIN CLL-321 trial, where pirtobrutinib significantly improved PFS compared with investigator’s choice therapy, reducing the risk of disease progression or death by approximately 46% [82], with median PFS approaching 20 months in heavily pretreated populations [83]. Additional comparative data from BRUIN CLL-314 suggest response rates comparable to or slightly higher than those of ibrutinib (~87% vs. 78.5%) in both treatment-naïve and relapsed settings [84], although the clinical significance of incremental ORR differences remains uncertain in the absence of mature overall survival (OS) data. These findings reinforce the evolving role of noncovalent BTK inhibition as a strategy designed specifically to address acquired resistance associated with earlier-generation covalent BTKi.

The safety profile of pirtobrutinib appears favorable across available studies. Rates of class-associated toxicities such as atrial fibrillation, bleeding, and hypertension appear lower than those observed with first-generation BTKi [85,86,87]. Common adverse events include fatigue, diarrhea, and neutropenia, which are generally low grade and manageable with supportive care [33,88,89]. This improved tolerability is likely related to enhanced kinase selectivity and reduced off-target inhibition [90]. Nonetheless, class-wide effects such as platelet dysfunction and infection risk remain relevant considerations in BTK inhibitor therapy [91,92]. Longer follow-up is required to more fully characterize long-term and cumulative toxicities, particularly in the context of continuous therapy, although patient-reported outcomes suggest acceptable tolerability over time [93]. From a comparative perspective, the toxicity profile of pirtobrutinib differs substantially from that observed with antibody–drug conjugates or HDAC inhibitors, where peripheral neuropathy or hematologic toxicity may more significantly limit prolonged treatment exposure.

In the context of NHL, pirtobrutinib is being integrated into an increasingly complex therapeutic landscape that includes CAR-T cell therapies, bispecific antibodies, and other targeted agents. Its activity in patients previously treated with CAR-T therapy or stem cell transplantation suggests a potential role in salvage or bridging strategies [94]. However, prospective comparative data remain limited, and optimal sequencing relative to cellular therapies is not yet clearly defined. Importantly, while kinase inhibition offers mechanistically precise targeting of B-cell receptor signaling, adaptive resistance through pathway reactivation or bypass signaling remains a persistent therapeutic challenge.

Combination strategies represent a key area of ongoing investigation and reflect a rational attempt to enhance response depth and overcome resistance mechanisms [95,96,97,98]. Combination-based therapeutic strategies are increasingly being explored to enhance the depth and durability of response associated with targeted NHL therapies. Early-phase studies are evaluating pirtobrutinib in combination with anti-CD20 monoclonal antibodies, BCL2 inhibitors such as venetoclax, and emerging immunotherapeutic agents [95]. For example, pirtobrutinib has demonstrated promising activity in combination with venetoclax, supported by the complementary inhibition of B-cell receptor signaling and BCL-2-mediated antiapoptotic pathways, which may synergistically promote lymphoma cell death and overcome resistance mechanisms observed with BTK inhibitor monotherapy. These approaches are biologically supported by complementary targeting of survival pathways (e.g., BTK–BCL2 axis) and modulation of tumor–immune interactions. Early-phase clinical studies evaluating BTK inhibitor–venetoclax combinations have reported encouraging activity in relapsed or refractory B-cell malignancies, including improved response depth and potential enhancement of minimal residual disease negativity, supporting continued exploration of chemotherapy-free targeted regimens [96,97,98]. However, metabolic adaptation and persistence mechanisms, including reprogramming of cellular energetics, may influence resistance and response durability [99]. While preliminary data suggest potential synergy, robust randomized evidence is still lacking, and overlapping toxicities as well as optimal scheduling require careful evaluation [100]. Early clinical experience further suggests that rationally designed combination regimens may improve response durability and therapeutic efficacy, although larger randomized studies remain necessary to define their optimal positioning within evolving NHL treatment algorithms. Compared with epigenetic therapies such as belinostat, these kinase inhibitor–based combinations may offer more biomarker-defined mechanistic targeting, although long-term resistance suppression remains uncertain.

Outside hematologic malignancies, there is currently no established role for pirtobrutinib in solid tumors such as breast cancer. Although BTK signaling has been implicated in tumor microenvironment modulation and immune regulation, clinical evidence supporting BTK inhibition in solid tumors remains limited and inconclusive [101,102].

From a broader perspective, pirtobrutinib exemplifies a rational approach to overcoming targeted therapy resistance through structure-guided drug design. Its ability to inhibit both wild-type and mutant BTK—including common resistance-associated C481 substitutions—positions it as a promising therapeutic option for patients who have progressed on prior BTKi [103,104]. However, emerging resistance mechanisms—including additional BTK mutations and activation of alternative signaling pathways—highlight the need for continued research into combination strategies and biomarker-driven patient selection [105]. In contrast to antibody–drug conjugates, which rely on antigen expression and intracellular payload delivery, kinase inhibition provides continuous suppression of oncogenic signaling but may be more vulnerable to adaptive pathway rewiring and clonal evolution over time.

Importantly, much of the current evidence relies on surrogate endpoints such as ORR and PFS. While these endpoints provide early signals of efficacy, their translation into OS benefit remains to be fully established [106]. Variability in trial design, patient populations, and comparator regimens further complicates cross-study comparisons, underscoring the need for cautious interpretation and the importance of randomized confirmatory trials. Moreover, differences in disease biology across NHL subtypes limit direct extrapolation of efficacy outcomes between indolent and aggressive lymphomas.

Pirtobrutinib represents an important advance in the management of B-cell malignancies, particularly in the relapsed or refractory NHL setting. Its activity in BTK inhibitor–resistant disease, favorable safety profile, and emerging role across multiple disease subtypes—including MCL, FL, MZL, and RT—support its growing relevance within precision oncology. Nevertheless, the level of supporting evidence varies across disease settings, and several conclusions continue to rely primarily on early-phase or single-arm studies [107,108,109].

Kinase inhibition with pirtobrutinib represents a mechanistically precise and clinically active therapeutic paradigm, particularly valuable for overcoming acquired resistance to earlier BTKi, although questions regarding long-term durability, optimal sequencing, and resistance evolution remain incompletely resolved. Compared with antibody–drug conjugate therapy and epigenetic modulation, pirtobrutinib currently benefits from a relatively more mature biomarker rationale and broader integration into contemporary NHL treatment algorithms, but continued randomized validation remains essential. Ongoing randomized trials, longer follow-up, and biomarker-guided strategies will therefore be essential to more clearly define its optimal positioning within evolving treatment algorithms and increasingly combination-based therapeutic paradigms.

Table 1 summarizes treatment-emergent adverse events (TEAEs) and their management strategies for pirtobrutinib, while Table 2 outlines the major pivotal clinical trials and selected emerging studies of pirtobrutinib in NHL.

## 3. Brentuximab Vedotin

Brentuximab vedotin is an ADC composed of a chimeric anti-CD30 monoclonal antibody linked to the microtubule-disrupting agent MMAE, representing a targeted cytotoxic strategy designed to selectively deliver chemotherapy to CD30-expressing malignant cells [113,114]. CD30, a member of the tumor necrosis factor receptor superfamily, is highly expressed in several NHL subtypes, including systemic anaplastic large cell lymphoma (sALCL) and subsets of other PTCLs, while exhibiting more limited expression in normal tissues [115,116]. CD30 is also characteristically expressed in classical Hodgkin lymphoma, which contributed to the early clinical development of brentuximab vedotin; however, its current role in NHL is particularly relevant in CD30-positive T-cell lymphomas and selected B-cell lymphoma settings [117]. At the same time, the biologic and prognostic role of CD30 appears context-dependent and may vary according to histology, cell of origin, and microenvironmental features, particularly in DLBCL [118].

**Figure 3:**
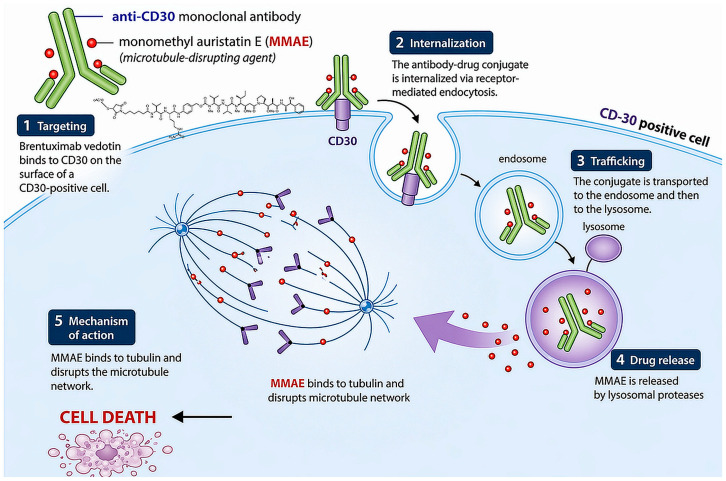
Brentuximab vedotin mechanism of action, adapted from Ref. [124]. Original schematic illustration created by the authors based on the mechanism described in [124]. Brentuximab vedotin targets CD30-positive cells, is internalized and trafficked to lysosomes, where MMAE is released to disrupt microtubules, leading to cell cycle arrest and apoptotic cell death. All abbreviations employed are defined in the text in the Abbreviations Section.

Mechanistically, brentuximab vedotin binds to CD30 on the tumor cell surface, undergoes internalization, and traffics to lysosomes, where the protease-cleavable linker releases MMAE into the cytoplasm [119]. MMAE subsequently binds to tubulin, disrupting microtubule dynamics, inducing cell-cycle arrest at the G2/M phase, and ultimately triggering apoptotic cell death [120,121]. In addition to direct cytotoxicity, a bystander effect has been described, whereby MMAE diffuses into adjacent cells, potentially enhancing antitumor activity in heterogeneous tumors with variable CD30 expression [122]. Furthermore, emerging evidence suggests immunomodulatory effects, including enhanced antigen presentation and modulation of the tumor microenvironment, although the clinical relevance of these mechanisms remains incompletely defined [123]. These multimodal properties likely contribute to the broader-than-expected activity of brentuximab vedotin in diseases with variable CD30 expression, while also complicating the interpretation of CD30 as a strict predictive biomarker. Figure 3 depicts the mechanism of action of brentuximab vedotin.

The initial clinical development of brentuximab vedotin included pivotal phase II studies in relapsed or refractory Hodgkin lymphoma and sALCL. In Hodgkin lymphoma, early trials demonstrated substantial activity after autologous stem cell transplantation (ASCT), supporting regulatory development and establishing proof of concept for CD30-targeted ADC therapy [125,126,127,128,129]. More directly relevant to NHL, the phase II Study 2 (NCT00866047) in relapsed or refractory systemic ALCL demonstrated ORRs of 86%, CR rates of 57%, and a median DoR of 12.6 months in heavily pretreated patients [35]. These findings established brentuximab vedotin as a major therapeutic advance in relapsed CD30-positive NHL, particularly PTCL. However, the single-arm design of these early studies and the absence of comparator arms necessitate cautious interpretation, particularly regarding long-term survival benefit. Compared with kinase inhibitors or epigenetic therapies, brentuximab vedotin demonstrated relatively high response rates in biomarker-selected populations, although the durability of benefit remains influenced by disease biology, antigen heterogeneity, and prior treatment exposure.

Subsequent randomized trials further expanded the role of brentuximab vedotin in NHL. The phase III ECHELON-2 (NCT01777152) trial compared brentuximab vedotin combined with cyclophosphamide, doxorubicin, and prednisone (BV-CHP) versus standard CHOP in previously untreated CD30-positive PTCL. This study demonstrated significant improvements in both PFS and OS, establishing BV-CHP as a new standard of care in this setting [36]. Extended follow-up confirmed durable clinical benefit, with sustained PFS and OS advantages at 5 years [36,130]. Importantly, the magnitude of benefit was greatest in systemic ALCL, emphasizing the biologic heterogeneity of PTCL and raising questions regarding the generalizability of these findings to subtypes with lower or more heterogeneous CD30 expression. Contemporary expert analyses further stress that the efficacy of “CHOP-plus” approaches is not uniform across PTCL histologies and should not be interpreted as biologically equivalent across the entire PTCL spectrum [131,132]. Additional subtype-specific molecular features may further influence therapeutic response [133]. Emerging evidence further suggests that predictive biomarkers beyond CD30 expression alone may contribute to therapeutic responsiveness, including tumor microenvironment composition, immune-cell infiltration patterns, cytokine-mediated signaling, and variability in intracellular payload processing and MMAE sensitivity. These observations support the concept that CD30 expression should be interpreted within a broader biologic and immunologic context rather than as an isolated binary biomarker. In contrast to BTK inhibition, where resistance is frequently mediated by pathway-specific molecular alterations, resistance to ADC therapy may additionally involve antigen modulation, impaired internalization, altered payload trafficking, or microenvironment-mediated survival signaling.

Hodgkin lymphoma studies such as ECHELON-1 and AETHERA additionally demonstrated the broader clinical activity of brentuximab vedotin in CD30-positive lymphoid malignancies [117,134]. These trials provided important contextual evidence supporting the therapeutic validity of CD30 targeting, although their direct applicability to NHL treatment algorithms remains limited. Consequently, current NHL-focused interest centers primarily on PTCL, CTCL, and selected LBCL settings rather than Hodgkin lymphoma-specific frontline or post-transplant strategies [135,136,137,138,139].

In relapsed or refractory NHL, particularly PTCL and other CD30-positive entities, brentuximab vedotin has shown meaningful activity both as monotherapy and in combination regimens. The phase III ALCANZA (NCT01578499) trial in CD30-positive cutaneous T-cell lymphoma (CTCL) demonstrated superior durable response rates compared with physician’s choice, confirming improved disease control in this population [140]. Final analyses further confirmed the durability of response and consistent clinical benefit across subgroups, with a manageable safety profile and no new safety signals [140,141]. Additionally, the phase III ECHELON-3 (NCT04404283) trial extended the role of brentuximab vedotin into relapsed/refractory LBCL, where its combination with lenalidomide and rituximab significantly improved OS, as well as PFS and ORR, in transplant- or CAR-T–ineligible patients [142]. These findings were supported by subsequent analyses confirming efficacy across clinically relevant subgroups and a manageable safety profile [142,143]. Together, these studies highlight an expanding role beyond classical CD30-high T-cell lymphomas, although patient selection remains critical and the biologic basis for benefit in lower-CD30-expression settings remains incompletely resolved. Contemporary lymphoma management frameworks increasingly position brentuximab vedotin within broader NHL treatment algorithms that also incorporate CAR-T therapy, bispecific antibodies, and subtype-specific salvage strategies [144]. Compared with epigenetic therapies such as belinostat, brentuximab vedotin currently benefits from stronger randomized clinical evidence and clearer integration into frontline treatment paradigms, although its applicability remains dependent on antigen expression and subtype-specific biology.

Additional NHL-focused studies evaluating combinations with bendamustine, gemcitabine-based regimens, platinum-containing salvage therapy, and checkpoint inhibitors have reported encouraging response rates, although these findings are derived primarily from early-phase trials with limited sample sizes [145,146,147,148,149,150,151]. However, the heterogeneity of PTCL and variability in CD30 expression complicate interpretation of these data, underscoring the need for biomarker-informed approaches to optimize patient selection. Emerging translational investigations are increasingly evaluating immune-related biomarkers, tumor-associated macrophage composition, T-cell exhaustion markers, and spatial immune architecture as potential determinants of response and resistance. Such findings may eventually facilitate more refined biologic stratification and individualized therapeutic sequencing in CD30-positive NHL.

Combination strategies involving brentuximab vedotin and immune checkpoint inhibitors have also generated interest due to potentially complementary mechanisms of action [152]. Combination-based therapeutic strategies are increasingly being explored to enhance the depth and durability of response associated with targeted NHL therapies. In particular, brentuximab vedotin has shown encouraging activity in combination with immune checkpoint inhibition, including nivolumab-based regimens, where immunogenic cell death induced by the antibody–drug conjugate may augment antitumor immune responses and potentially enhance T-cell–mediated cytotoxicity. Mechanistically, MMAE-mediated tumor cell death may promote antigen release and immune activation, thereby providing a biologic rationale for synergy with PD-1 blockade. Although much of the initial clinical experience originated in Hodgkin lymphoma [153,154], emerging studies in B-cell lymphomas suggest that these combinations may broaden activity beyond classical CD30-high diseases [155]. Early-phase clinical studies have reported encouraging response rates and durable remissions in selected relapsed or refractory lymphoma populations, supporting continued investigation of these immunotherapy-based combination approaches. Nevertheless, additive toxicity, cost, and the absence of mature comparative NHL-specific data continue to limit routine adoption across all settings. These findings illustrate the broader evolution of ADC therapy from simple targeted cytotoxic delivery toward increasingly immunologically integrated therapeutic strategies.

The safety profile of brentuximab vedotin is well characterized and largely consistent across studies. The most common adverse events include peripheral neuropathy, neutropenia, fatigue, and gastrointestinal symptoms [134]. Peripheral neuropathy is dose-dependent and may be cumulative, often necessitating dose modification or discontinuation [156]. Hematologic toxicity, particularly neutropenia, is also common, especially in combination regimens, and may require growth factor support [157]. Rare but serious adverse events include progressive multifocal leukoencephalopathy and pancreatitis, although these are infrequent [158]. Real-world reports continue to underscore the clinical importance of neurologic toxicity, particularly in patients with pre-existing risk factors or treatment-related vulnerabilities [159]. In special populations, including older or frail patients and those with major comorbidities, tolerability considerations may substantially affect regimen selection and dose intensity [160,161]. Overall, the toxicity profile is manageable but requires careful monitoring, particularly in the context of prolonged therapy or multidrug combinations. Importantly, the toxicity spectrum of ADC therapy differs mechanistically from that of kinase inhibitors or HDAC inhibitors, with peripheral neuropathy representing a particularly distinctive limitation of MMAE-containing ADCs.

In the broader context of precision oncology, brentuximab vedotin exemplifies the successful application of ADC technology in hematologic malignancies. Its efficacy is closely linked to CD30 expression, although the threshold of expression required for clinical benefit remains uncertain. Notably, responses have been observed even in tumors with low or heterogeneous CD30 expression, suggesting that additional mechanisms, including bystander effects and microenvironmental interactions, may contribute to therapeutic activity [162]. This observation complicates the use of CD30 as a strict predictive biomarker and highlights the need for more refined biomarker strategies. Emerging evidence suggests that additional biomarkers beyond CD30 expression alone may influence responsiveness to brentuximab vedotin, including tumor microenvironment composition, immune-cell infiltration patterns, and variability in intracellular payload processing [15,19,20,41,118,131]. It also suggests that CD30 expression should be interpreted not simply as a binary marker, but within a broader biologic framework that includes spatial heterogeneity, immune contexture, and disease subtype. Although these biomarkers remain investigational, they may contribute to improved therapeutic stratification and resistance prediction in future NHL treatment paradigms. Relative to kinase inhibition, which targets intracellular signaling pathways, ADC therapy offers a therapeutically attractive strategy capable of combining biologic specificity with direct cytotoxic delivery, although dependence on antigen expression and payload-related toxicity remain important limitations.

In non-hematologic malignancies, including breast cancer, brentuximab vedotin has not demonstrated a defined clinical role. While CD30 expression has been reported in a minority of solid tumors, including certain breast cancer subtypes, clinical evidence supporting the efficacy of CD30-targeted therapy in this context remains limited and largely investigational [163]. Consequently, its use remains confined to CD30-positive lymphoid malignancies.

From a critical perspective, the development of brentuximab vedotin illustrates both the strengths and limitations of targeted cytotoxic strategies. Randomized NHL-focused trials such as ECHELON-2, ALCANZA, and ECHELON-3 have clearly established its value in selected settings [164,165,166], yet variability in CD30 expression, underlying disease biology, and treatment context limits straightforward generalization across NHL subtypes. Similarly, although numerous combination strategies appear promising, many remain supported mainly by phase I/II datasets, and their long-term impact on survival, toxicity burden, and therapeutic sequencing is not yet fully defined. Compared with pirtobrutinib, which primarily addresses resistance through continuous kinase suppression, or belinostat, which exerts broader epigenetic reprogramming effects, brentuximab vedotin occupies an intermediate therapeutic paradigm characterized by high initial activity but persistent dependence on antigen biology and payload tolerability.

Furthermore, the reliance on surrogate endpoints such as PFS in several key trials underscores the need for longer follow-up to determine whether observed benefits consistently translate into meaningful improvements in OS [167]. Differences in trial design, patient populations, prior therapies, and comparator regimens further complicate cross-study comparisons, necessitating cautious interpretation of the evidence base. Some NHL settings now also require comparison against rapidly evolving standards incorporating checkpoint inhibitors, CAR-T therapy, or other novel immunotherapeutic platforms, which may further reshape the relative positioning of brentuximab vedotin.

Brentuximab vedotin represents a major advance in the treatment of CD30-positive NHL, with demonstrated efficacy across multiple disease settings and lines of therapy, particularly in systemic ALCL, other CD30-positive PTCLs, CTCL, and selected LBCL populations. Its integration into frontline regimens, salvage strategies, and combination approaches reflects its versatility as a targeted therapeutic agent. However, ongoing studies are needed to refine patient selection, optimize combination strategies, better define its role relative to competing immunotherapeutic approaches, and further clarify its place within evolving, biomarker-driven NHL treatment algorithms [166,167,168,169,170,171,172]. Population-based analyses from the brentuximab vedotin era and continuing real-world studies will also be important for understanding long-term outcomes beyond clinical trial populations [173].

Antibody–drug conjugate therapy with brentuximab vedotin represents one of the most clinically validated targeted approaches currently available in NHL, particularly within CD30-positive T-cell lymphomas, although challenges related to resistance biology, biomarker refinement, and toxicity optimization continue to define important areas for future investigation.

Table 3 summarizes TEAEs and their management strategies for brentuximab vedotin, while Table 4 outlines the major pivotal clinical trials and selected emerging studies of brentuximab vedotin in NHL.

## 4. Belinostat

Belinostat is a pan-HDAC inhibitor belonging to the class of epigenetic modulators that alter gene expression through reversible acetylation of histone and non-histone proteins, thereby influencing chromatin structure and transcriptional activity [176,177]. HDAC enzymes play a central role in regulating gene expression by removing acetyl groups from lysine residues on histones, leading to chromatin condensation and transcriptional repression. Dysregulation of HDAC activity has been implicated in lymphomagenesis, particularly in T-cell lymphomas, where aberrant epigenetic silencing contributes to malignant transformation and disease progression [178,179,180]. By inhibiting multiple HDAC isoforms, belinostat promotes histone acetylation, chromatin relaxation, reactivation of tumor suppressor genes, and induction of cell-cycle arrest and apoptosis in malignant cells [181,182,183]. This mechanistic breadth distinguishes belinostat from pathway-specific kinase inhibitors, but it also contributes to a broader and less predictable spectrum of biologic effects [184,185].

**Figure 4:**
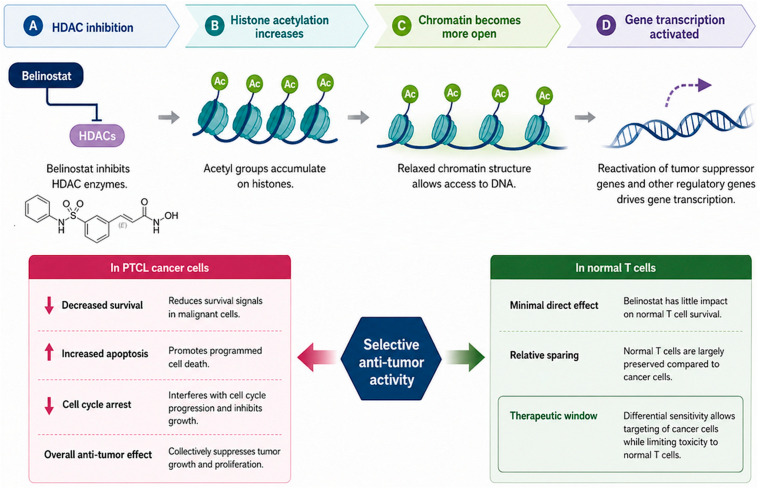
Belinostat mechanism of action. Original schematic illustration created by the authors based on the mechanism described in [191]. Belinostat inhibits HDAC activity, increasing histone acetylation and chromatin accessibility, which promotes pro-apoptotic gene expression and induces cell cycle arrest and apoptosis in peripheral T-cell lymphoma cells. All abbreviations employed are defined in the text in the Abbreviations Section.

Beyond its direct epigenetic effects, belinostat influences cellular processes including modulation of cell-cycle regulators, inhibition of angiogenesis, and alteration of immune responses within the tumor microenvironment [186]. Preclinical studies suggest that HDAC inhibition may enhance antigen presentation and increase susceptibility to immune-mediated killing, providing a rationale for combination strategies with immunotherapies [187]. However, the pleiotropic nature of HDAC inhibition may narrow the therapeutic window and complicate translation into consistently durable clinical benefit. Broader reviews of HDAC inhibitor development in lymphoma have emphasized this duality, highlighting both mechanistic promise and the difficulty of identifying patients most likely to benefit [188,189,190]. Figure 4 presents the mechanism of action of belinostat.

The clinical development of belinostat in NHL was established primarily in relapsed or refractory PTCL, a heterogeneous group of aggressive malignancies with historically poor outcomes and limited treatment options. The pivotal phase II BELIEF trial (NCT00865969) evaluated belinostat in patients with relapsed or refractory PTCL (n = 129), including PTCL-NOS, angioimmunoblastic T-cell lymphoma (AITL), and ALK-negative ALCL, and demonstrated an ORR of approximately 26%, including CRs in a subset of patients [40]. The median DoR was 13.6 months, indicating that durable remissions may be achievable in selected patients [192]. Responses were observed across multiple PTCL subtypes, suggesting activity beyond a single histologic category. A post hoc subgroup analysis also demonstrated maintained clinical activity in patients with baseline thrombocytopenia, with manageable hematologic toxicity [192]. These findings supported the regulatory approval of belinostat in relapsed or refractory PTCL [193,194,195]. Compared with kinase inhibition or antibody–drug conjugate therapy, belinostat represents a broader and less biomarker-defined therapeutic paradigm, in which clinical activity reflects modulation of transcriptional and epigenetic programs rather than inhibition of a single dominant oncogenic driver.

Importantly, however, belinostat should be distinguished from agents supported by randomized phase III or broader comparative evidence. In contrast to therapies such as brentuximab vedotin in CD30-positive PTCL, belinostat remains supported primarily by a single-arm phase II registration study, with no randomized phase III trials to date [40,192]. This substantially limits the strength of comparative evidence and constrains its precise positioning within modern treatment algorithms. Recent policy-focused analyses have also used belinostat as an example of the broader challenge posed by accelerated approvals that are not followed rapidly by confirmatory trials, raising important questions regarding evidence maturity and long-term regulatory confidence [196]. From an integrative perspective, this evidentiary gap places belinostat in a less firmly established position than brentuximab vedotin, which benefits from randomized NHL-focused trial data, or pirtobrutinib, which is increasingly supported by subtype-specific prospective studies.

Interpretation of the BELIEF trial therefore requires caution. Response rates, while clinically meaningful in a difficult-to-treat population, remain modest compared with some newer targeted, antibody-based, or cellular approaches [40]. Moreover, the heterogeneity of PTCL—including major differences in molecular drivers, epigenetic architecture, and microenvironmental dependencies—likely contributes to variable treatment response [178,179,180,197]. Contemporary reviews of PTCL biology and management increasingly stress that this heterogeneity is central to therapeutic decision-making and may partly explain why epigenetic therapies demonstrate activity in some patients yet remain difficult to optimize across broader populations [198,199,200,201]. The absence of validated biomarker-driven patient selection further limits the ability to identify the subgroups most likely to benefit from HDAC inhibition. Emerging translational studies are increasingly investigating whether epigenetic signatures, histone acetylation profiles, chromatin-remodeling alterations, and tumor microenvironment characteristics may eventually support more refined therapeutic stratification. In particular, recurrent alterations involving epigenetic regulators and transcriptional programs in PTCL subtypes such as AITL may partly explain differential sensitivity to HDAC inhibition, although these observations remain investigational and are not yet clinically validated.

Earlier phase I studies, such as NCT00274651, established the safety, tolerability, and recommended dosing schedule of belinostat in patients with advanced NHL, demonstrating preliminary antitumor activity across T-cell lymphoma subtypes [202]. These foundational data informed subsequent phase II development and supported the dosing strategy used in relapsed/refractory PTCL. Later clinical characterization suggested that certain subtypes, particularly AITL, may derive relatively greater benefit, although these observations remain exploratory rather than definitive [203]. This subtype signal is biologically plausible given the epigenetic vulnerability of selected PTCL entities, but it remains less clinically actionable than CD30-directed selection for brentuximab vedotin or BTK pathway targeting for pirtobrutinib.

Subsequent studies have explored belinostat in combination with chemotherapy and other targeted agents in an effort to improve efficacy. Early-phase trials combining belinostat with CHOP (Bel-CHOP), including the phase I/II NCT01839097 study, demonstrated encouraging response rates, with ORRs exceeding 80% in some PTCL cohorts and an acceptable safety profile [204]. While these results suggest potential synergy between epigenetic modulation and cytotoxic chemotherapy, the lack of randomized phase III data limits definitive conclusions regarding survival benefit. Differences in trial design, patient selection, and endpoint definitions further complicate comparisons with established regimens. Thus, these data support further investigation of HDAC inhibitor-based combinations but do not yet establish belinostat-containing regimens as broadly evidence-equivalent to randomized standards.

Additional combination strategies have evaluated belinostat with cytotoxic and epigenetic agents. Studies combining belinostat with carboplatin and paclitaxel (NCT00421889) demonstrated manageable toxicity and preliminary antitumor activity in relapsed or refractory aggressive lymphomas [205,206,207]. Similarly, dual epigenetic-targeting strategies, such as belinostat combined with azacitidine (NCT00351975), have shown early evidence of synergistic activity, supporting the rationale for epigenetic combination approaches [205,208]. Broader HDAC-based combination strategies remain under investigation in early-phase studies, with limited but evolving clinical data [209,210,211]. Preclinical work has further suggested synergy between HDAC inhibition and proteasome inhibition, PLK1 inhibition, Src-pathway targeting, geranylgeranyl transferase inhibition, and other biologically complementary approaches [212,213,214,215]. These findings are mechanistically intriguing, but most have not yet translated into robust clinical evidence. In comparison with pirtobrutinib-based or brentuximab vedotin-based combinations, belinostat combinations remain more exploratory, reflecting both the complexity of epigenetic modulation and the difficulty of defining optimal partner agents.

The safety profile of belinostat is generally consistent with other HDAC inhibitors but appears comparatively favorable in certain respects. Common adverse events include nausea, fatigue, anemia, and thrombocytopenia, which are typically manageable with supportive care and dose adjustments [216,217]. Notably, belinostat has been associated with a lower incidence of severe thrombocytopenia than some other HDAC inhibitors, which may facilitate combination therapy [218]. However, hematologic toxicity remains a significant concern, particularly in heavily pretreated patients, and requires close monitoring. Clinical overviews of PTCL pharmacotherapy have generally positioned belinostat as one of the more tolerable epigenetic agents in this setting, although with efficacy that remains moderate and supported primarily by non-randomized evidence [219]. Compared with brentuximab vedotin, belinostat does not have the same characteristic burden of cumulative peripheral neuropathy; however, its more modest response rates and less clearly defined predictive framework limit its precision-oncology impact.

Cardiac toxicity, including QT prolongation, has been reported with HDAC inhibitors, although the incidence with belinostat appears relatively low [220,221]. Nevertheless, baseline and periodic electrocardiographic monitoring is recommended, particularly in patients with preexisting cardiac risk factors. Additionally, hepatic metabolism via UGT1A1 necessitates caution in patients with impaired liver function, and dose adjustments may be required [222,223]. Pharmacogenetic considerations may therefore be relevant in individualized dosing, particularly in patients with reduced UGT1A1 activity [224].

In the broader context of precision oncology, belinostat represents a distinct therapeutic paradigm focused on epigenetic reprogramming rather than direct targeting of oncogenic signaling pathways. While this approach offers the theoretical advantage of modulating multiple oncogenic processes simultaneously, it also introduces challenges related to specificity, response predictability, and mechanistic attribution [225]. Unlike kinase inhibitors, where treatment selection may be anchored to a defined actionable alteration, there are currently no validated predictive biomarkers for routine clinical selection of patients most likely to benefit from belinostat. This remains a major limitation to its integration into biomarker-driven treatment algorithms. Emerging investigations are exploring whether epigenetic signatures, histone acetylation patterns, molecular alterations affecting chromatin remodeling pathways, and immune-related microenvironmental features may eventually improve prediction of therapeutic responsiveness. Similarly, tumor microenvironment interactions and immune-cell infiltration patterns are increasingly being investigated as potential modulators of sensitivity and resistance to HDAC inhibition. Although these biomarkers remain investigational, they may contribute to improved therapeutic stratification and resistance prediction in future NHL treatment paradigms. Even when candidate biomarkers are proposed, however, their relevance may be disease-specific and not directly translatable across lymphoma subtypes [226].

In non-hematologic malignancies, including breast cancer, belinostat has been evaluated in early-phase studies, but clinical activity has been modest and inconsistent [227]. Although epigenetic dysregulation is recognized across many solid tumors, the translation of HDAC inhibition into meaningful clinical benefit has been limited, and its role outside hematologic malignancies remains investigational.

From a critical standpoint, the clinical development of belinostat illustrates both the therapeutic promise and the limitations of epigenetic strategies in lymphoma. Although durable responses can be achieved in a subset of patients with PTCL, ORRs remain modest, and the absence of randomized comparative trials precludes definitive conclusions regarding its relative efficacy within the broader treatment landscape [228]. Interpretation of available data is further complicated by heterogeneity in trial populations, prior lines of therapy, and underlying disease biology, which together limit cross-study comparability and reinforce the need for more refined, biomarker-driven patient selection. Real-world and anecdotal experience confirms that prolonged disease control is possible in selected patients, but such cases remain exceptional rather than predictive of routine outcomes [229]. Compared with pirtobrutinib and brentuximab vedotin, belinostat currently occupies a more selective and later-line role, particularly for patients in whom tolerability, prior treatment exposure, and lack of alternative options shape therapeutic decision-making.

The reliance on surrogate endpoints such as ORR in pivotal studies reinforces the importance of longer-term follow-up to better define the impact of belinostat on PFS and OS outcomes [163,230]. In parallel, the incorporation of belinostat into combination regimens introduces additional complexity related to cumulative toxicity, optimal sequencing, and cost-effectiveness, all of which must be balanced against potential improvements in clinical efficacy. More broadly, outcomes analyses in PTCL suggest that participation in clinical trials and access to newer agents may improve survival, but the relative contribution of belinostat specifically remains difficult to isolate from the expanding therapeutic landscape [231].

Ongoing research is therefore increasingly focused on identifying molecular and epigenetic biomarkers predictive of response to HDAC inhibition, as well as on developing rational combinations with immunotherapy and other targeted agents to enhance antitumor activity and overcome resistance mechanisms [232]. Preclinical studies also indicate that resistance to HDAC inhibition may create new therapeutic vulnerabilities, suggesting that adaptive resistance itself could become a basis for rational salvage strategies [233]. Within this evolving framework, belinostat remains a mechanistically distinct but comparatively less strongly validated option in relapsed or refractory PTCL, exerting its effects through HDAC inhibition and subsequent modulation of chromatin architecture and gene transcription [234,235]. This rationale is supported by the epigenetically dysregulated landscape of PTCL, characterized by recurrent alterations in chromatin-modifying genes and substantial transcriptional heterogeneity across molecular subtypes [236,237]. In contrast to the more target-defined paradigms represented by BTK inhibition and CD30-directed ADC therapy, belinostat provides a broader epigenetic intervention that may be particularly relevant in biologically heterogeneous PTCL, but this same breadth also complicates patient selection and resistance prediction.

Clinically, belinostat is associated with a manageable safety profile and favorable tolerability relative to intensive chemotherapy, supporting its use particularly in heavily pretreated or more vulnerable patient populations [238,239]. At the same time, the rapidly evolving NHL therapeutic landscape underscores the potential of HDAC inhibitor-based combination strategies to augment immune-mediated antitumor responses and address resistance pathways [240,241]. Despite these advances, the overall efficacy of belinostat as monotherapy remains limited, with modest response rates and a persistent lack of validated predictive biomarkers to guide optimal patient selection [238,242]. Moreover, the absence of randomized phase III evidence continues to constrain its definitive positioning within treatment algorithms, underscoring the need for prospective, biomarker-driven studies to more precisely define its role in precision oncology frameworks [239,242].

Epigenetic modulation with belinostat represents a biologically compelling but clinically less mature NHL therapeutic paradigm compared with kinase inhibition and antibody–drug conjugate therapy, with its future value likely dependent on biomarker refinement, rational combinations, and prospective validation in molecularly defined PTCL subgroups.

Table 5 summarizes TEAEs and their management strategies for belinostat, while Table 6 outlines the major pivotal clinical trials and selected key clinical studies of belinostat in NHL.

## 5. Advancing Frontiers in Non-Hodgkin Lymphoma: Future Directions and Emerging Approaches

The therapeutic landscape of NHL is undergoing substantial transformation driven by advances in molecular biology, immunotherapy, and precision oncology. Increasingly, NHL is understood not as a single entity but as a spectrum of molecularly defined diseases shaped by distinct genetic, epigenetic, and microenvironmental determinants [244,245]. Although targeted agents such as pirtobrutinib, brentuximab vedotin, and belinostat have improved outcomes in selected settings, their optimal integration remains constrained by incomplete biomarker frameworks, disease heterogeneity, evolving resistance patterns, and the rapidly changing therapeutic landscape shaped by CAR-T-cell therapy and bispecific antibodies [5,246]. Consequently, the transition from empiric treatment algorithms toward truly biologically guided strategies remains an ongoing, rather than fully realized, objective [33,247].

Advances in molecular profiling technologies—including NGS, single-cell transcriptomics, and spatial analysis—have enabled increasingly granular characterization of tumor heterogeneity [99,248,249]. These approaches are refining disease classification by linking genomic alterations with functional and spatial context, thereby improving stratification and exposing potentially targetable vulnerabilities [113,250]. Emerging genomic and transcriptomic studies in FL, transformed FL, and DLBCL further illustrate how integrated profiling may reveal therapeutically relevant subgroups that are not fully captured by conventional histopathologic classification alone [251]. Molecular subtyping in DLBCL exemplifies this progress; however, its clinical implementation remains limited by variability in assay standardization, accessibility, and incomplete prospective validation [36,252]. Similarly, ctDNA and minimal residual disease (MRD) monitoring may help optimize the clinical positioning and sequencing of targeted therapies such as pirtobrutinib, brentuximab vedotin, and belinostat by enabling earlier detection of resistance or relapse, although methodological and clinical validation challenges currently restrict routine adoption [253,254].

Therapeutic resistance remains a central limitation of targeted strategies. Clonal evolution under treatment pressure frequently leads to the emergence of resistant subpopulations, as observed with covalent BTKi and mutations such as BTK C481S [255,256,257,258]. Non-covalent BTKi, including pirtobrutinib, address some of these resistance mechanisms and demonstrate activity in heavily pretreated populations; however, the durability of response and their precise positioning within treatment sequences require further clarification [259,260]. More broadly, efforts to develop pathway-driven therapies in NHL increasingly recognize that resistance is rarely mediated by a single lesion and instead reflects adaptive pathway redundancy and metabolic plasticity. This is evident in emerging preclinical work targeting convergent survival programs, including PI3Kδ- and PPARα-linked signaling in FL [261]. Combination approaches involving pirtobrutinib and agents targeting complementary pathways such as BCL2 or PI3K are biologically rational, although their clinical value must be balanced against greater toxicity, cost, and treatment complexity [262,263,264].

Combination strategies remain central to improving outcomes with the three selected agents, particularly given the redundancy and cross-talk of oncogenic signaling pathways in NHL [265,266]. Brentuximab vedotin has shown significant efficacy across CD30-positive lymphomas, especially when incorporated into combination regimens [267,268]. Nevertheless, broader application remains limited by antigen heterogeneity, uncertainty regarding optimal CD30 thresholds, and toxicity considerations, particularly in heavily pretreated or frail patients. Ongoing work continues to highlight the complexity of CD30 as a biomarker and the need for better methods to evaluate expression and predict benefit across biologically diverse NHL subtypes [269]. Studies combining brentuximab vedotin with immune checkpoint inhibitors or other immunotherapeutic approaches aim to enhance efficacy, although long-term safety, comparative benefit, and optimal sequencing remain to be established [172,270]. Similarly, belinostat offers a mechanistically distinct epigenetic strategy, but its clinical utility remains constrained by modest response rates and the absence of randomized comparative data [271,272]. Epigenetic therapies retain biologic promise, particularly in rational combinations with immunotherapy or targeted agents, yet the lack of validated predictive biomarkers remains a major barrier to broader clinical integration [273,274].

Emerging immunotherapeutic platforms may further influence the positioning of pirtobrutinib, brentuximab vedotin, and belinostat within future NHL treatment algorithms. CAR-T therapy and bispecific antibodies have demonstrated substantial activity in relapsed and refractory B-cell lymphomas [275,276,277,278,279,280,281,282,283,284,285,286,287,288,289,290,291]; however, their relevance to this discussion lies primarily in how they may complement, precede, or follow targeted therapies in increasingly individualized treatment sequences. Cellular and T-cell-engaging immunotherapies are already reshaping standards of care in several aggressive lymphoma settings, potentially altering the sequencing and clinical utilization of targeted small molecules and antibody–drug conjugates. In this context, ongoing studies are evaluating whether agents such as pirtobrutinib can enhance immune-based strategies or serve as bridging therapies, while brentuximab vedotin-containing regimens continue to be explored alongside immunotherapeutic combinations [172,270]. Nevertheless, despite encouraging clinical activity, the long-term positioning of pirtobrutinib, brentuximab vedotin, and belinostat within NHL treatment algorithms remains incompletely defined, particularly in the rapidly evolving era of CAR-T-cell therapy and bispecific antibodies. Important questions regarding sequencing, cumulative toxicity, resistance, comparative effectiveness, and patient selection therefore remain unresolved.

A deeper understanding of tumor evolution is essential for improving long-term disease control. Tumor plasticity enables adaptation through genetic and epigenetic mechanisms as well as through interactions with the tumor microenvironment [292]. While longitudinal monitoring with ctDNA offers potential insights into these dynamics, its integration into routine clinical decision-making remains limited by technical, analytical, and interpretive challenges [293]. Future strategies involving pirtobrutinib, brentuximab vedotin, and belinostat will likely depend on improved biomarker-driven selection, more effective resistance monitoring, and rational sequencing approaches designed to delay therapeutic escape and better integrate targeted therapies with emerging immune-based modalities.

Artificial intelligence (AI) and machine learning are also being explored as tools to refine treatment stratification and biomarker integration in NHL [294,295,296]. Although systematic review data in DLBCL suggest potential utility for predictive modeling, methodological heterogeneity and limited external validation continue to restrict clinical implementation [297]. Similarly, adaptive and biomarker-driven trial designs may facilitate more efficient evaluation of targeted combinations and sequencing strategies involving pirtobrutinib, brentuximab vedotin, and belinostat, although increasing trial complexity may also create challenges in interpretation and broader implementation [298,299].

A key objective in NHL management is the development of chemotherapy-sparing approaches that incorporate targeted therapies and immunotherapeutic combinations [300,301]. In this regard, agents such as pirtobrutinib, brentuximab vedotin, and belinostat exemplify the broader shift toward biologically driven treatment strategies, particularly for older or medically unfit patients. However, real-world tolerability, cumulative toxicity, treatment adherence, and cost-effectiveness remain important considerations [302,303]. Longer follow-up and comparative effectiveness studies are still required to clarify whether these agents can consistently replace conventional chemotherapy or are best integrated within sequential and combination-based treatment paradigms that increasingly include CAR-T-cell therapy and bispecific antibodies [304,305,306,307,308].

Finally, progress in NHL will depend not only on therapeutic innovation but also on improvements in clinical trial design and equitable access to care. Adaptive and platform-based trials are enabling more efficient evaluation of novel agents, particularly in molecularly defined populations [309,310,311]. However, disparities in access to molecular diagnostics, targeted therapies, and advanced immunotherapeutic approaches remain a major challenge [312,313,314]. The future clinical impact of pirtobrutinib, brentuximab vedotin, and belinostat will therefore depend not only on biologic efficacy, but also on improved biomarker integration, optimized sequencing strategies, comparative evaluation relative to cellular immunotherapies, and broader real-world accessibility [315].

In summary, the future role of pirtobrutinib, brentuximab vedotin, and belinostat in NHL management will likely depend on improved molecular stratification, rational combination approaches, and more precise sequencing within evolving treatment algorithms. Although emerging technologies and immunotherapeutic strategies continue to expand therapeutic possibilities, their greatest relevance lies in how they may enhance the clinical integration, resistance management, and biomarker-driven application of these three targeted agents. Future studies will therefore need to clarify the optimal integration of these therapies within increasingly complex NHL treatment frameworks that incorporate CAR-T-cell therapy, bispecific antibodies, biomarker-guided sequencing, and combination-based precision approaches. Substantial clinical and logistical challenges nevertheless remain before these precision-based strategies can be fully optimized in routine NHL care.

**Figure 5:**
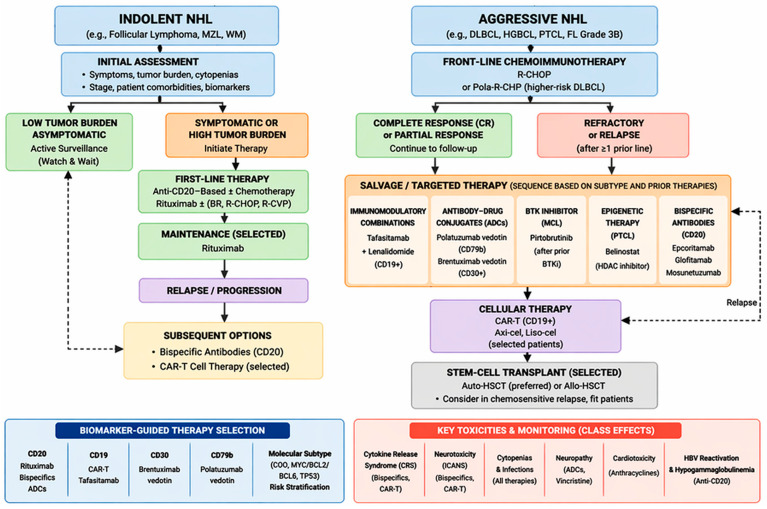
Therapeutic pathways and treatment algorithms in non-Hodgkin lymphoma integrating biomarkers and treatment sequencing. The presented algorithm constitutes an original, author-developed interpretative synthesis of current clinical evidence and pivotal trial findings, aligned with major NCCN and ESMO principles, including biomarker-guided therapeutic selection and treatment sequencing according to prior therapeutic exposure and mechanisms of resistance. The figure was independently created for this manuscript and does not reproduce, replicate, or directly adapt any previously published guideline, algorithm, or source material. Elements reflecting emerging or investigational therapeutic strategies are clearly indicated and should be interpreted within the context of continuously evolving clinical evidence and ongoing translational research. All abbreviations employed are defined in the text in the Abbreviations Section.

Table 7 provides an overview of the comparative characteristics of pirtobrutinib, brentuximab vedotin, and belinostat in precision-based therapeutic strategies for NHL; Table 8 outlines contemporary NHL management, including indications, treatment regimens, biomarkers, toxicity profiles, and levels of supporting evidence; while Figure 5 illustrates therapeutic pathways and treatment algorithms integrating biomarker-driven selection and treatment sequencing in NHL.

## 6. Conclusions

NHL represents a biologically heterogeneous group of malignancies in which precision oncology is increasingly reshaping therapeutic decision-making by aligning treatment strategies with disease biology. The clinical development of pirtobrutinib, brentuximab vedotin, and belinostat illustrates three complementary therapeutic paradigms—targeting intracellular signaling, delivering selective cytotoxic payloads, and modulating epigenetic regulation—highlighting the ability to intervene at multiple levels of lymphomagenesis.

Collectively, these agents also demonstrate that therapeutic efficacy depends not only on target expression, but also on broader biologic context, including molecular subtype, tumor microenvironment, prior treatment exposure, and mechanisms of adaptive resistance. The experience with pirtobrutinib highlights the importance of overcoming acquired resistance associated with covalent BTK inhibition, particularly BTK C481 alterations, while also illustrating the growing relevance of treatment sequencing in heavily pretreated B-cell NHL. Brentuximab vedotin emphasizes both the therapeutic value and the limitations of biomarker-directed ADC therapy, as CD30 expression alone does not fully predict clinical benefit and must be interpreted within a broader biologic framework. In contrast, belinostat illustrates the challenges associated with epigenetic therapies in PTCL, where biologic rationale and regulatory approval have preceded the development of robust predictive biomarkers and randomized comparative evidence.

These agents further underscore the importance of balancing efficacy with toxicity and long-term treatment feasibility. Peripheral neuropathy associated with brentuximab vedotin, class-specific toxicities linked to BTK inhibition, and the hematologic and systemic adverse effects observed with HDAC inhibition all influence treatment selection, sequencing, and combination strategies in clinical practice. At the same time, differences in evidence maturity across these therapies remain highly relevant. While agents such as brentuximab vedotin are supported by randomized phase III data in selected NHL settings, belinostat continues to rely primarily on single-arm phase II evidence, highlighting the uneven pace at which targeted therapies advance from biologic promise to fully validated standards of care.

Importantly, the evolving therapeutic landscape of NHL increasingly requires adaptable and biomarker-informed strategies capable of responding to clonal evolution and therapeutic resistance. The integration of molecular profiling, rational combination approaches, and improved resistance monitoring may help optimize the positioning of these agents within future treatment algorithms. However, substantial challenges remain regarding biomarker validation, comparative effectiveness, sequencing after novel immunotherapies, cumulative toxicity, and equitable access to advanced therapies.

Overall, pirtobrutinib, brentuximab vedotin, and belinostat together illustrate both the progress and the ongoing limitations of precision oncology in NHL. Their development highlights critical lessons regarding biomarker selection, resistance mechanisms, toxicity management, and the necessity of aligning therapeutic intensity and sequencing with disease biology and patient-specific factors. Continued efforts to refine molecular stratification, validate predictive biomarkers, and generate higher-level comparative evidence will be essential to more fully integrate targeted therapies into coherent, biologically informed NHL treatment frameworks.

## Figures and Tables

**Table 1:** TEAEs and management strategies for pirtobrutinib according to [33,72,110]. All abbreviations employed are defined in the text in the Abbreviations Section.

TEAE	Frequency/Severity	Timing/Clinical Features	Recommended Management
Infections	Common; severity variable, including grade ≥ 3 events	May occur at any time during treatment; includes respiratory and viral infections, with higher clinical relevance in heavily pretreated or immunocompromised patients	Monitor closely for fever and infection symptoms; initiate prompt diagnostic workup and antimicrobial therapy as indicated; consider dose interruption for severe infection.
Hemorrhage/bruising/contusion	Common overall; major hemorrhage uncommon	Usually low-grade bruising or contusion, but serious bleeding can occur; risk may increase with anticoagulants, antiplatelet agents, or thrombocytopenia	Review bleeding risk and concomitant medications; monitor clinically; interrupt treatment for significant bleeding and resume only after risk–benefit reassessment.
Cytopenias (neutropenia, anemia, thrombocytopenia)	Common; may be grade ≥ 3, especially neutropenia	Often detected during routine monitoring; may contribute to infection risk, fatigue, or bleeding	Perform serial CBC monitoring; use dose interruption or reduction for clinically significant cytopenias; provide growth factor support or transfusion as appropriate.
Fatigue	Common; usually grade 1–2	Frequently reported across BRUIN cohorts; may accumulate over time and may be multifactorial	Evaluate reversible causes, optimize hydration and nutrition, encourage activity pacing, and consider dose modification for persistent grade ≥ 3 fatigue.
Gastrointestinal toxicity (diarrhea, nausea, abdominal symptoms)	Common; mostly grade 1–2	Usually early or intermittent during therapy; generally manageable with supportive care	Use hydration, dietary adjustment, and antidiarrheal/antiemetic therapy as needed; consider dose modification for persistent or severe symptoms.
Musculoskeletal and constitutional symptoms (musculoskeletal pain, arthralgia, headache)	Common; usually low grade	May emerge during ongoing therapy; typically non-limiting but can affect quality of life	Symptomatic treatment with analgesics and supportive care; reassess if persistent or function-limiting.
Dyspnea/cough	Common; usually grade 1–2, occasionally clinically significant	May reflect treatment-related symptoms, infection, anemia, or underlying cardiopulmonary disease	Evaluate for infection, anemia, and disease progression; manage symptomatically; interrupt therapy if severe or unexplained.
Cardiac arrhythmias (including atrial fibrillation/flutter)	Uncommon; grade ≥ 3 events relatively infrequent	May occur during treatment, particularly in patients with prior cardiac disease or risk factors	Assess baseline cardiovascular history; monitor clinically for palpitations or rhythm disturbance; interrupt and manage according to severity.
Rash and other cutaneous events	Less common; usually mild to moderate	Typically develops during treatment exposure and is often manageable	Use emollients, antihistamines, or topical corticosteroids when needed; interrupt treatment for severe skin toxicity.
Laboratory abnormalities/biochemical changes	Variable; may include grade ≥ 3 hematologic laboratory abnormalities	Usually detected on routine monitoring rather than by symptoms alone	Regular laboratory surveillance and correction of reversible abnormalities; manage according to severity and associated clinical findings.
Rare serious events (severe infection, major bleeding, cardiac events)	Rare but clinically important	More relevant in older, heavily pretreated, or comorbid populations	Prompt evaluation, treatment interruption, and specialist-directed management when severe toxicity is suspected.

**Table 2:** Major pivotal clinical trials and selected emerging studies of pirtobrutinib in non-Hodgkin lymphoma. All abbreviations employed are defined in the text in the Abbreviations Section.

Trial/Study	Population	Cancer Setting	Design	Combination	Key Findings	Inclusion/ Eligibility Criteria
BRUIN phase 1/2 (NCT03740529) [33]	R/R B-cell malignancies; key NHL cohort: MCL after prior covalent BTKi	R/R NHL (primarily MCL)	Multicenter, open-label, phase 1/2	Monotherapy	ORR ~52% in cBTKi-pretreated MCL; durable responses	≥1 prior therapy; prior covalent BTKi required for MCL cohort
BRUIN MCL registration cohort [111]	n = 120 R/R MCL; median ~3 prior lines	R/R MCL after ≥2 prior lines incl. BTKi	Open-label, single-arm	Monotherapy	ORR 50%, CR 13%, median DoR 8.3 months; basis for approval	Prior BTKi required; majority refractory
BRUIN MCL-321 (NCT04662255) [78]	Previously treated, BTKi-naïve MCL	R/R MCL (earlier-line)	Global, randomized, phase III	Monotherapy vs. investigator’s choice BTKi	Ongoing confirmatory trial; primary endpoint: PFS	≥1 prior therapy; no prior BTKi
BRUIN (follicular lymphoma cohort) [74]	R/R FL; median ~3 prior lines	R/R indolent NHL (FL)	Phase 1/2 cohort analysis	Monotherapy	ORR ~52%, CR ~16.7%; median DoR ~10 months	Relapsed/refractory FL
BRUIN (marginal zone lymphoma cohort) [75]	R/R MZL	R/R indolent NHL (MZL)	Phase 1/2 cohort analysis	Monotherapy	ORR ~55%; durable responses	Prior therapy required
BRUIN (Richter transformation cohort) [112]	RT (DLBCL-type)	Aggressive lymphoma	Phase 1/2 subgroup analysis	Monotherapy	Encouraging activity in high-risk population	Prior therapies allowed
GATE1 (NCT06522386) [95]	Previously untreated MCL	Frontline MCL	Phase II	Pirtobrutinib + rituximab + venetoclax	Ongoing triplet targeted strategy	Untreated MCL
Pirtobrutinib + venetoclax (NCT05529069) [96]	Relapsed/refractory MCL	R/R MCL	Phase II	Combination	Ongoing; BTK–BCL2 targeting	Prior therapy required
Pirtobrutinib + rituximab (NCT06263491) [97]	Newly diagnosed low/intermediate-risk MCL	Frontline MCL	Phase II	Combination	Ongoing chemo-free strategy	Untreated MCL
Pirtobrutinib + glofitamab (NCT06252675) [98]	Relapsed/refractory mantle cell lymphoma	R/R MCL	Phase II	Combination	Ongoing exploratory regimen	Relapsed/refractory disease

BRUIN-derived publications [33,74,75,107,108] correctly share NCT03740529.

**Table 3:** TEAEs and management strategies for brentuximab vedotin according to [35,174,175]. All abbreviations employed are defined in the text in the Abbreviations Section.

TEAE	Frequency/Severity	Timing/Clinical Features	Recommended Management
Peripheral neuropathy	Common; cumulative; usually grade 1–2, but may reach grade ≥ 3	A key toxicity across NHL studies. In ECHELON-2, peripheral neuropathy occurred in 52% and was predominantly sensory, with median onset at 2 months; in ECHELON-3, it occurred in 27%, with median onset at 3 months.	Regular neurologic assessment; dose delay, reduction, or discontinuation for persistent or severe neuropathy; supportive care for neuropathic symptoms.
Hematologic toxicity (neutropenia, anemia, thrombocytopenia, febrile neutropenia)	Common; neutropenia may be severe, especially in combination regimens	In ECHELON-2, febrile neutropenia occurred in 18% with A + CHP versus 15% with CHOP. In ECHELON-3, dose delays were commonly driven by neutropenia (23%) and thrombocytopenia (8%).	CBC monitoring before each cycle; growth factor support when indicated; dose modification; transfusion or infection-directed management as needed; urgent treatment of febrile neutropenia.
Gastrointestinal toxicity (nausea, diarrhea, constipation, vomiting, abdominal pain, decreased appetite)	Common; mostly grade 1–2	In ECHELON-2, nausea and diarrhea were among the adverse reactions seen more often with A + CHP; in ECHELON-3, diarrhea occurred in 31% of patients receiving BV-based therapy.	Antiemetics, hydration, dietary support, bowel regimen, and dose adjustment for persistent or severe symptoms.
Fatigue/asthenia	Common; generally low grade, occasionally grade ≥ 3	Fatigue is a recurrent toxicity in BV-treated NHL populations. In ECHELON-2, fatigue/asthenia was more frequent with A + CHP than CHOP; in ECHELON-3, fatigue occurred in 46% of BV-treated patients.	Assess reversible contributors, optimize nutrition and hydration, encourage activity pacing, and consider dose modification for persistent grade ≥ 3 fatigue.
Pyrexia and infections	Common to serious; severity variable	In ECHELON-2, pyrexia was more frequent with A + CHP. In ECHELON-3, serious adverse reactions included pneumonia (21%), COVID-19 (13%), sepsis (9%), and febrile neutropenia (7%).	Prompt infection workup, antimicrobial therapy when indicated, supportive care, and treatment interruption for serious infectious events.
Respiratory toxicity/pulmonary events	Less common but potentially severe	May present with dyspnea, cough, pneumonitis, interstitial changes, or infectious pneumonia. Pulmonary toxicity is included in the prescribing information warnings; pneumonia was a major serious AE in ECHELON-3.	Evaluate urgently to distinguish infection, drug toxicity, and disease progression; interrupt treatment; provide supportive care; discontinue in severe or recurrent cases.
Cutaneous toxicity (rash, pruritus)	Common; usually mild to moderate	Rash is among the common adverse reactions reported with BV-containing regimens, including ECHELON-3.	Emollients, antihistamines, topical corticosteroids if needed, and treatment interruption for severe skin toxicity.
Infusion-related reactions	Uncommon to less common; occasionally severe	Typically occur during or shortly after infusion; manifestations may include chills, nausea, dyspnea, pruritus, pyrexia, or cough.	Slow or interrupt infusion, provide symptomatic treatment, consider premedication in selected patients, and discontinue for severe recurrent reactions.
Hepatotoxicity	Uncommon but clinically relevant; occasionally severe	Can occur after the first dose or on rechallenge, with transaminase and/or bilirubin elevation.	Monitor liver function tests before and during therapy; delay, reduce, or discontinue treatment for clinically significant hepatotoxicity.
Serious neurologic and opportunistic events (including PML)	Rare but potentially life-threatening	The prescribing information carries a boxed warning for JC virus-associated PML; onset may occur at variable times after treatment initiation.	Immediate evaluation of new neurologic symptoms; hold therapy if PML is suspected; permanently discontinue if confirmed.
Rare serious systemic events (pancreatitis, severe dermatologic reactions, gastrointestinal complications, hyperglycemia)	Rare but clinically important	These events are less frequent but are highlighted in the prescribing information as important safety concerns.	Prompt evaluation and supportive management; interrupt or discontinue treatment according to severity and clinical context.

**Table 4:** Major pivotal clinical trials and studies of brentuximab vedotin in non-Hodgkin lymphoma. All abbreviations employed are defined in the text in the Abbreviations Section.

Trial/Study	Population	Cancer Setting	Design	Combination	Key Findings	Inclusion/ Eligibility Criteria
Study 2 (NCT00866047) [35]	Relapsed/refractory sALCL (n = 58)	Relapsed/refractory	Phase II, open-label, single-arm, multicenter	Brentuximab vedotin	ORR 86%, CR 57%; median DoR 12.6 months; durable responses in heavily pretreated patients	Histologically confirmed sALCL; ≥1 prior systemic therapy; relapsed or refractory disease; prior auto-HSCT allowed
ECHELON-2 (NCT01777152) [36]	Previously untreated CD30+ PTCL, including sALCL, PTCL-NOS, AITL (n = 452)	Front-line	Phase III, randomized, double-blind, active-controlled	Brentuximab vedotin + CHP vs. CHOP	Improved PFS (48.2 vs. 20.8 months; HR 0.71) and OS benefit; established BV + CHP as standard in CD30+ PTCL	CD30 expression ≥10%; untreated PTCL; ECOG 0–1; adequate organ function
ALCANZA (NCT01578499) [140]	CD30+ cutaneous T-cell lymphoma (CTCL), including mycosis fungoides and pcALCL (n = 131)	Relapsed/refractory	Phase III, randomized, open-label, multicenter	Brentuximab vedotin vs. physician’s choice (methotrexate or bexarotene)	Durable response ≥4 months: 56.3% vs. 12.5%; superior disease control vs. standard options	Prior systemic therapy; CD30+ MF or pcALCL; candidates for systemic treatment
ECHELON-3 (NCT04404283) [142]	Relapsed/refractory LBCL, including DLBCL and HGBL (n = 230)	Relapsed/refractory	Phase III, randomized, double-blind, placebo-controlled	Brentuximab vedotin + lenalidomide + rituximab vs. placebo + lenalidomide + rituximab	Improved OS (13.8 vs. 8.5 months; HR 0.63); PFS and ORR benefit (64.3% vs. 41.5%)	≥2 prior lines; transplant/CAR-T ineligible; ECOG 0–2; measurable disease

ALCANZA is usually grouped under NHL because CTCL/pcALCL are T-cell NHL subtypes.

**Table 5:** TEAEs and management strategies for belinostat according to [40,243]. All abbreviations employed are defined in the text in the Abbreviations Section.

TEAE	Frequency/Severity	Timing/Clinical Features	Recommended Management
Gastrointestinal toxicity (nausea, vomiting, decreased appetite, diarrhea, constipation, abdominal pain)	Common; mostly grade 1–2	Usually early during treatment; may reduce oral intake and impair tolerability	Antiemetics, hydration, dietary support, bowel regimen, and dose delay/reduction for persistent or severe symptoms
Fatigue	Common; usually grade 1–2, occasionally grade ≥ 3	May emerge early and accumulate over cycles; often multifactorial	Assess reversible causes, optimize supportive care, and consider dose modification for persistent grade ≥ 3 fatigue
Hematologic toxicity (anemia, thrombocytopenia, neutropenia, febrile neutropenia)	Common; clinically significant in some patients	May occur during early or subsequent cycles, especially in heavily pretreated patients	Regular CBC monitoring, dose interruption/reduction, transfusional support, and prompt management of febrile neutropenia or severe cytopenias
Pyrexia and infections	Common to less common; severity variable	Fever may be isolated or associated with infection, particularly in cytopenic patients	Prompt evaluation for infection, supportive care, antimicrobial therapy when indicated, and treatment interruption for serious events
Respiratory and fluid-related events (dyspnea, cough, peripheral edema)	Common; occasionally grade ≥ 3	May reflect treatment effect, infection, anemia, comorbidity, or disease progression	Clinical assessment, supportive management, and treatment interruption with further workup for severe symptoms
Cutaneous toxicity (rash, pruritus)	Usually mild to moderate	Develops during treatment exposure; generally manageable	Emollients, antihistamines, topical corticosteroids if needed, and treatment interruption for severe skin toxicity
Laboratory abnormalities (hypokalemia, elevated LDH, other biochemical changes)	Usually mild to moderate; occasionally clinically relevant	Often detected on routine monitoring	Serial laboratory monitoring, electrolyte replacement, and correction of contributing factors
Cardiovascular events (QT prolongation, hypotension, dizziness)	Less common but clinically relevant	May occur during infusion or early cycles; risk increased by electrolyte imbalance or cardiac comorbidity	Review concomitant drugs, correct electrolytes, monitor at-risk patients, and interrupt treatment for symptomatic or severe events
Hepatotoxicity	Uncommon but potentially severe	May present with elevated liver enzymes or clinical liver dysfunction	Monitor liver function tests before and during therapy; interrupt or discontinue treatment for significant hepatic toxicity
Rare serious events (tumor lysis syndrome, multisystem toxicity)	Rare but potentially life-threatening	More likely early in treatment, particularly in patients with high tumor burden	Risk assessment, prophylaxis in high-risk patients, close monitoring, and immediate supportive management with treatment interruption if suspected
Infusion-related and other less frequent general events (chills, headache, phlebitis, infusion-site pain)	Usually low grade	Typically occur during or shortly after infusion	Symptomatic treatment, local supportive care, and infusion interruption/slowing if clinically indicated

**Table 6:** Major pivotal clinical trials and key clinical studies of belinostat in non-Hodgkin lymphoma. All abbreviations employed are defined in the text in the Abbreviations Section.

Trial/Study	Population	Cancer Setting	Design	Combination	Key Findings	Inclusion/ Eligibility Criteria
BELIEF (NCT00865969) [40]	Relapsed/refractory PTCL (n = 129), including PTCL-NOS, AITL, ALK−ALCL; subset with thrombocytopenia (n ≈ 38)	Relapsed/refractory	Phase II, open-label, single-arm, multicenter (with post hoc subgroup analysis)	Belinostat monotherapy	ORR 25.8%, CR 10.8%; median DoR 13.6 months; clinically meaningful activity in heavily pretreated PTCL; subset analysis: maintained activity (~15–20% ORR) in patients with thrombocytopenia with manageable hematologic toxicity	≥1 prior systemic therapy; histologically confirmed PTCL; measurable disease; ECOG 0–2; adequate organ function; platelet count < 100 × 10^9^/L permitted in subset
Bel-CHOP Phase I/II combination study (NCT01839097) [204]	Relapsed/refractory aggressive NHL, including PTCL and DLBCL	Relapsed/refractory	Phase I/II, open-label, dose-escalation and expansion	Belinostat + CHOP (Bel-CHOP)	Acceptable safety profile; high response rates in PTCL cohorts (ORR > 80% in some subsets); supports combination strategies	Untreated or relapsed aggressive NHL; adequate organ function; ECOG 0–2; dose-limiting toxicity assessment in escalation phase
Early phase study (NCT00274651) [202]	Relapsed/refractory T-cell lymphoma and other NHL subtypes	Relapsed/refractory	Phase I, open-label, dose-escalation	Belinostat monotherapy	Demonstrated safety, tolerability, and preliminary antitumor activity; established recommended dosing schedule	Advanced NHL after prior therapy; measurable disease; adequate marrow, hepatic, and renal function
Belinostat + carboplatin/paclitaxel (NCT00421889) [207]	Relapsed/refractory aggressive lymphomas (including PTCL and DLBCL)	Relapsed/refractory	Phase I/II	Combination (chemotherapy backbone)	Manageable toxicity; preliminary antitumor activity	Relapsed/refractory NHL; adequate organ function
Belinostat + azacitidine (NCT00351975) [208]	T-cell lymphomas (PTCL)	Relapsed/refractory	Phase I/II	Combination (epigenetic therapy)	Early evidence of synergistic activity via dual epigenetic modulation	Relapsed/refractory PTCL; measurable disease
Belinostat-based HDAC combinations (various early-phase studies) [209,210,211]	Advanced lymphomas (various NHL subtypes)	Relapsed/refractory	Early phase	Combination (epigenetic/targeted)	Exploratory strategies; limited clinical data	Advanced NHL; prior therapies allowed

**Table 7:** Comparative characteristics of pirtobrutinib, brentuximab vedotin, and belinostat in precision therapy for non-Hodgkin lymphoma according to [33,35,36,40,107,203,316,317]. All abbreviations employed are defined in the text in the Abbreviations Section.

Agent	Therapeutic Class	Primary Molecular Target	Main NHL Indications	Biomarker Relevance	Key Resistance Mechanisms	Major Toxicities	Evidence Maturity	Potential Combination Strategies
Pirtobrutinib	Noncovalent Bruton tyrosine kinase (BTK) inhibitor	BTK within B-cell receptor (BCR) signaling pathway	Relapsed/refractory mantle cell lymphoma (MCL); other relapsed B-cell NHL subtypes under investigation	BTK pathway dependence; prior covalent BTK inhibitor exposure; B-cell molecular phenotype	BTK-independent signaling escape; PLCγ2 alterations; clonal evolution; microenvironment-mediated survival signaling	Neutropenia, fatigue, infections, diarrhea, bleeding events, atrial fibrillation (less frequent than with earlier BTK inhibitors)	Primarily early-phase and registration-supporting evidence; ongoing confirmatory studies	Venetoclax; anti-CD20 monoclonal antibodies; bispecific antibodies; CAR-T sequencing approaches
Brentuximab Vedotin	CD30-directed antibody–drug conjugate (ADC)	CD30 surface antigen linked to monomethyl auristatin E (MMAE) payload	Systemic anaplastic large-cell lymphoma (sALCL); CD30-positive peripheral T-cell lymphoma (PTCL); selected CD30-positive diffuse large B-cell lymphoma (DLBCL)	CD30 expression level and distribution; tumor microenvironment characteristics	CD30 downregulation or heterogeneity; impaired ADC internalization; drug efflux mechanisms; MMAE resistance	Peripheral neuropathy, neutropenia, fatigue, infusion-related reactions, infections	Randomized and practice-changing evidence in selected CD30-positive lymphomas	Nivolumab; chemotherapy regimens (e.g., CHP); bispecific antibodies; immune-based combinations
Belinostat	Histone deacetylase (HDAC) inhibitor	Class I and II HDAC enzymes involved in epigenetic regulation	Relapsed/refractory peripheral T-cell lymphoma (PTCL)	Epigenetic dysregulation; histone acetylation profiles; exploratory chromatin-remodeling markers	Epigenetic adaptation; compensatory survival signaling; tumor heterogeneity; limited biomarker-guided selection	Nausea, fatigue, thrombocytopenia, anemia, infections, hepatic enzyme elevation	Primarily single-arm and early-phase evidence; limited randomized confirmatory data	Hypomethylating agents; chemotherapy combinations; immunomodulatory agents; exploratory targeted combinations

**Table 8:** Contemporary management of non-Hodgkin lymphoma: clinically focused overview of current treatment strategies, including indications, representative regimens, biomarkers, toxicity considerations, and supporting evidence. All abbreviations employed are defined in the text in the Abbreviations Section.

Modality	Indication/When Used	Example Regimens/Agents	Key Evidence	Biomarkers/Toxicity
Active surveillance	Asymptomatic, low–tumor burden indolent NHL (e.g., FL)	Observation	NCCN/ESMO guidance [1,2,318]	No immediate biomarker-driven trigger; risk of delayed treatment; requires close monitoring
Radiotherapy (ISRT)	Limited-stage disease; palliation of localized symptoms	Involved-site RT	Practice guidelines [1,2,318]	Site-dependent toxicity; marrow suppression; organ-specific late effects
Front-line chemoimmunotherapy (aggressive B-cell NHL)	Newly diagnosed DLBCL and aggressive B-cell NHL	R-CHOP; pola-R-CHP	POLARIX; historical R-CHOP trials [319,320]	COO subtype, MYC/BCL2/BCL6; toxicities: cytopenias, cardiotoxicity, neuropathy
Anti-CD20–based therapy (indolent NHL)	First-line or relapsed follicular and CD20 + NHL	Rituximab monotherapy; BR; rituximab-chemotherapy followed by maintenance in selected cases	StiL; BRIGHT [321,322]	CD20 expression; infections, hypogammaglobulinemia, HBV reactivation
BTKi (MCL)	Relapsed/refractory MCL	Pirtobrutinib	BRUIN study [33]	MCL subtype; bleeding, infections, atrial arrhythmias (class effect)
Immunomodulatory combinations	Transplant-ineligible relapsed/refractory DLBCL	Tafasitamab + lenalidomide	L-MIND [323]	CD19 expression; neutropenia, infections, thromboembolism
Bispecific antibodies	Relapsed/refractory B-cell NHL (DLBCL, FL)	Epcoritamab; glofitamab; mosunetuzumab	EPCORE NHL-1; NP30179; GO29781 [324,325,326]	CD20 expression; CRS, ICANS, cytopenias
ADCs	Front-line (selected) or relapsed NHL; CD30+ PTCL	Polatuzumab vedotin; brentuximab vedotin; brentuximab vedotin + lenalidomide + rituximab in relapsed/refractory LBCL	POLARIX; ECHELON-2 [36,319]	CD79b (pola), CD30 (BV); neuropathy, cytopenias
Epigenetic therapy (HDAC inhibitors)	Relapsed/refractory PTCL	Belinostat	BELIEF trial [40]	No routine biomarker; myelosuppression, fatigue, hepatotoxicity
CAR-T cell therapy	Relapsed/refractory LBCL; early relapse or refractory disease	Axi-cel; liso-cel; other approved CD19 CAR-T products by subtype/line	ZUMA-1; TRANSCEND [327,328]	CD19 expression; CRS, ICANS, prolonged cytopenias
Stem-cell transplantation	Selected relapsed/refractory aggressive NHL or PTCL	Auto-HSCT; allo-HSCT in selected cases	Historical transplant data [1,2,318]	Fitness, disease status; TRM, GVHD (allo), infections

## Data Availability

No new data were created or analyzed in this study.

## Abbreviations

The following abbreviations are used in this manuscript:

Ac	acetyl group
ADC(s)	antibody–drug conjugate(s)
AE	adverse event
AITL	angioimmunoblastic T-cell lymphoma
ALCL	anaplastic large cell lymphoma
ALK−ALCL	anaplastic lymphoma kinase-negative anaplastic large cell lymphoma
Allo-HSCT	allogeneic hematopoietic stem cell transplantation
Auto-HSCT	autologous hematopoietic stem cell transplantation
Axi-cel	axicabtagene ciloleucel
BCR	B-cell receptor
Bel-CHOP	belinostat plus cyclophosphamide, doxorubicin, vincristine, prednisone
BR	bendamustine plus rituximab
BTK	Bruton tyrosine kinase
BTKi	Bruton tyrosine kinase inhibitor
BV	brentuximab vedotin
CAR-T	chimeric antigen receptor T-cell
Ca2+	calcium ions
CBC	complete blood count
CD	cluster of differentiation
CHOP	cyclophosphamide, doxorubicin, vincristine, prednisone
CHP	cyclophosphamide, doxorubicin, prednisone
COO	cell of origin
CR(s)	complete response(s)
CRS	cytokine release syndrome
cBTKi	covalent Bruton tyrosine kinase inhibitor
CTCL	cutaneous T-cell lymphoma
DAG	diacylglycerol
DLBCL	diffuse large B-cell lymphoma
DoR	duration of response
ECOG	Eastern Cooperative Oncology Group performance status
FL	follicular lymphoma
GVHD	graft-versus-host disease
HAT	histone acetyltransferase
HBV	hepatitis B virus
HDAC(s)	histone deacetylase(s)
HGBCL	high-grade B-cell lymphoma
HR	hazard ratio
ICANS	immune effector cell-associated neurotoxicity syndrome
Igα (CD79A)	immunoglobulin alpha chain
Igβ (CD79B)	immunoglobulin beta chain
IPI	International Prognostic Index
IP3	inositol 1,4,5-trisphosphate
IRC	independent review committee
ISRT	involved-site radiotherapy
LBCL	large B-cell lymphoma
Liso-cel	lisocabtagene maraleucel
LYN	LYN proto-oncogene, Src family tyrosine kinase
MCL	mantle cell lymphoma
MF	mycosis fungoides
MMAE	monomethyl auristatin E
MYC	MYC proto-oncogene
NCCN	National Comprehensive Cancer Network

## References

[1] NCCN Clinical Practice Guidelines in Oncology: B-Cell Lymphomas. National Comprehensive Cancer Network. https://www.nccn.org/guidelines/guidelines-detail?category=1&id=1480.

[2] NCCN Clinical Practice Guidelines in Oncology: T-Cell Lymphomas. National Comprehensive Cancer Network. https://www.nccn.org/guidelines/guidelines-detail?category=1&id=1483.

[3] Attygalle A.D., Chan J.K.C., Coupland S.E., Du M.-Q., Ferry J.A., de Jong D., Gratzinger D., Lim M.S., Naresh K.N., Nicolae A. (2024). The 5th Edition of the World Health Organization Classification of Mature Lymphoid and Stromal Tumors—An Overview and Update. Leuk. Lymphoma.

[4] Küppers R. (2005). Mechanisms of B-cell lymphoma pathogenesis. Nat. Rev. Cancer.

[5] Chapuy B., Cheng H., Watahiki A., Ducar M.D., Tan Y., Chen L., Roemer M.G.M., Ouyang J., Christie A.L., Zhang L. (2016). Diffuse Large B-Cell Lymphoma Patient-Derived Xenograft Models Capture the Molecular and Biological Heterogeneity of the Disease. Blood.

[6] Blombery P., de Jong D., Ferry J.A., Hsi E.D., Ondrejka S.L., Seymour J.F., Zamò A., Tzankov A. (2025). Closing the gap between biology and classification in splenic B-cell lymphomas. Histopathology.

[7] Tilly H., Gomes da Silva M., Vitolo U., Jack A., Meignan M., Lopez-Guillermo A., Walewski J., André M., Johnson P.W., Pfreundschuh M. (2015). Diffuse large B-cell lymphoma (DLBCL): ESMO clinical practice guidelines. Ann. Oncol..

[8] Coiffier B., Sarkozy C. (2016). Diffuse large B-cell lymphoma: R-CHOP failure—What to do?. Hematol. Am. Soc. Hematol. Educ. Program.

[9] Friedberg J.W. (2011). Relapsed/refractory diffuse large B-cell lymphoma. Hematol. Am. Soc. Hematol. Educ. Program.

[10] Tun A.M., Ansell S.M. (2020). Immunotherapy in Hodgkin and non-Hodgkin lymphoma: Innate, adaptive and targeted immunological strategies. Cancer Treat. Rev..

[11] Abramson J.S., Ghosh N., Smith S.M. (2020). ADCs, BiTEs, CARs, and small molecules: A new era of targeted therapy in non-Hodgkin lymphoma. Am. Soc. Clin. Oncol. Educ. Book.

[12] Sehn L.H., Salles G. (2021). Diffuse large B-cell lymphoma. N. Engl. J. Med..

[13] Lenz G., Staudt L.M. (2010). Aggressive lymphomas. N. Engl. J. Med..

[14] Gisselbrecht C., Van Den Neste E. (2018). How I manage patients with relapsed/refractory diffuse large B-cell lymphoma. Br. J. Haematol..

[15] Gong I.Y., Kuruvilla J. (2026). Management of relapsed/refractory diffuse large B-cell lymphoma in the era of novel agents and new approaches. Leuk. Lymphoma.

[16] Ernst M., Oeser A., Besiroglu B., Caro-Valenzuela J., Abd El Aziz M., Monsef I., Borchmann P., Estcourt L.J., Skoetz N., Goldkuhle M. (2021). Chimeric Antigen Receptor (CAR) T-Cell Therapy for People with Relapsed or Refractory Diffuse Large B-Cell Lymphoma. Cochrane Database Syst. Rev..

[17] Scott D.W., Gascoyne R.D. (2014). The tumour microenvironment in B-cell lymphomas. Nat. Rev. Cancer.

[18] Nowakowski G.S., Czuczman M.S. (2015). ABC, GCB, and double-hit diffuse large B-cell lymphoma: Does subtype make a difference in therapy selection?. Am. Soc. Clin. Oncol. Educ. Book.

[19] Makita S., Hosoba R., Tobinai K. (2020). Safety Considerations with Targeted Therapy Drugs for B-Cell Non-Hodgkin Lymphoma. Expert Opin. Drug Saf..

[20] Wang L., Qin W., Huo Y.J., Li X., Shi Q., Rasko J.E.J., Janin A., Zhao W.L. (2020). Advances in targeted therapy for malignant lymphoma. Signal Transduct. Target. Ther..

[21] Chaudhari J., Shah N.N. (2025). Targeted therapies and immunotherapies for diffuse large B-cell lymphoma. Cancers.

[22] Tiwari B., Afshan R., Sridhar S. (2025). Targeted therapies and resistance mechanisms in lymphoma: Current landscape and emerging solutions. Oncoscience.

[23] Cox M.C., Bocci G. (2022). Metronomic chemotherapy regimens and targeted therapies in non-Hodgkin lymphoma: The best of two worlds. Cancer Lett..

[24] Manji F., Puckrin R., Stewart D.A. (2021). Novel synthetic drugs for the treatment of non-Hodgkin lymphoma. Expert Opin. Pharmacother..

[25] Bühler M.M., Martin-Subero J.I., Pan-Hammarström Q., Campo E., Rosenquist R. (2022). Towards precision medicine in lymphoid malignancies. J. Intern. Med..

[26] Saeidnia M., Shokri M., Nourmohammadi H., Radmehr S., Babashahi M., Moradi M., Karimian M., Panji M., Motevaseli E. (2026). Advancing the diagnosis of non-Hodgkin lymphoma through next-generation sequencing in developing countries: An evaluation of progress—A narrative review. Health Sci. Rep..

[27] Autio M., Brück O., Pollari M., Karjalainen-Lindsberg M.-L., Beiske K., Mészaros Jørgen-sen J., Holte H., Pellinen T., Leivonen S.-K., Leppä S. (2025). Characterization of tumor microenvironment and cell interaction patterns in testicular and diffuse large B-cell lymphomas. Haematologica.

[28] García-Domínguez D.J., Hontecillas-Prieto L., Palazón-Carrión N., Jiménez-Cortegana C., Sánchez-Margalet V., de la Cruz-Merino L. (2022). Tumor immune microenvironment in lymphoma: Focus on epigenetics. Cancers.

[29] Byrd J.C., Harrington B., O’Brien S., Jones J.A., Schuh A., Devereux S., Chaves J., Wierda W.G., Awan F.T., Brown J.R. (2016). Acalabrutinib (ACP-196) in relapsed chronic lymphocytic leukemia. N. Engl. J. Med..

[30] Woyach J.A., Furman R.R., Liu T.-M., Ozer H.G., Zapatka M., Ruppert A.S., Xue L., Li D.H.-H., Steggerda S.M., Versele M. (2014). Resistance mechanisms for the Bruton’s tyro-sine kinase inhibitor ibrutinib. N. Engl. J. Med..

[31] Moldovianu A.-M., Stoia R., Vasilica M., Ursuleac I., Badelita S.N., Tomescu A.A., Pre-da O.D., Bardas A., Cirstea M., Coriu D. (2023). Real-world clinical outcomes and adverse events in patients with chronic lymphocytic leukemia treated with ibrutinib. Medicina.

[32] Telaraja D., Kasamon Y.L., Collazo J.S., Leong R., Wang K., Li P., Dahmane E., Yang Y., Earp J., Grimstein M. (2024). FDA approval summary: Pirtobrutinib for relapsed or refrac-tory mantle cell lymphoma. Clin. Cancer Res..

[33] Mato A.R., Shah N.N., Jurczak W., Cheah C.Y., Pagel J.M., Woyach J.A., Fakhri B., Eyre T.A., Lamanna N., Patel M.R. (2021). Pirtobrutinib in relapsed or refractory B-cell malignancies (BRUIN): A phase 1/2 study. Lancet.

[34] Caserta S., Martino E.A., Vigna E., Bruzzese A., Amodio N., Lucia E., Olivito V., Labanca C., Mendicino F., Morabito F. (2025). Bruton tyrosine kinase inhibitors in mantle cell lymphoma: What are the current options?. Eur. J. Haematol..

[35] Pro B., Advani R., Brice P., Bartlett N.L., Rosenblatt J.D., Illidge T., Matous J., Ramchandren R., Fanale M., Connors J.M. (2012). Brentuximab vedotin in relapsed or refractory systemic anaplastic large-cell lymphoma. J. Clin. Oncol..

[36] Horwitz S., O’Connor O.A., Pro B., Illidge T., Fanale M., Advani R., Bartlett N.L., Christensen J.H., Morschhauser F., Domingo-Domenech E. (2019). Erratum in: Department of Error: Brentuximab vedotin with chemotherapy for CD30-positive PTCL (ECHELON-2). Lancet.

[37] Sanders S., Stewart D.A. (2017). Targeting non-Hodgkin lymphoma with blinatumomab. Expert Opin. Biol. Ther..

[38] Younes A., Ansell S.M. (2016). Novel agents in Hodgkin lymphoma. Semin. Hematol..

[39] Letafati A., Chaijani R.M., Edalat F., Eslami N., Askari H., Askari F., Shirvani S., Talebzadeh H., Tarahomi M., MirKhani N. (2025). Advances in epigenetic treatment of adult T-cell leukemia/lymphoma. Clin. Epigenet..

[40] O’Connor O.A., Horwitz S., Masszi T., Van Hoof A., Brown P., Doorduijn J., Hess G., Jurczak W., Knoblauch P., Chawla S. (2015). Belinostat in Patients with Relapsed or Refractory Peripheral T-Cell Lymphoma: Results of the Pivotal Phase II BELIEF (CLN-19) Study. J. Clin. Oncol..

[41] Lam H.P.J., Amin F., Arulogun S.O., Gleeson M. (2025). Nodal peripheral T-cell lymphoma: Therapeutic challenges and future perspectives. Cancers.

[42] Meeuwes F.O., Serroukh Y.I.M., van der Poel M.W.M., Plattel W.J., Nijland M. (2025). Relapsed and refractory peripheral T-cell lymphoma: Treatment, challenges and future perspectives. Leuk. Lymphoma.

[43] June C.H., O’Connor R.S., Kawalekar O.U., Ghassemi S., Milone M.C. (2018). CAR T cell immunotherapy for human cancer. Science.

[44] Schuster S.J., Bishop M.R., Tam C.S., Waller E.K., Borchmann P., McGuirk J.P., Jäger U., Jaglowski S., Andreadis C., Westin J.R. (2019). Tisagenlecleucel in adult relapsed or refractory diffuse large B-cell lymphoma. N. Engl. J. Med..

[45] Pomykala K.L., Fendler W.P., Vermesh O., Umutlu L., Herrmann K., Seifert R. (2023). Molecular imaging of lymphoma: Future directions and perspectives. Semin. Nucl. Med..

[46] Roschewski M., Rossi D., Kurtz D.M., Alizadeh A.A., Wilson W.H. (2022). Circulating tumor DNA in lymphoma: Principles and future directions. Blood Cancer Discov..

[47] Kurtz D.M., Scherer F., Jin M.C., Soo J., Craig A.F.M., Esfahani M.S., Chabon J.J., Stehr H., Liu C.L., Tibshirani R. (2018). Circulating tumor DNA measurements as early outcome predictors in diffuse large B-cell lymphoma. J. Clin. Oncol..

[48] Roth J.A., Kratochvil D., Perkins K., Zhang W. (2026). Cost-efficiency modeling of conversion to biosimilar rituximab-based R-CHOP in diffuse large B-cell lymphoma in Medicare. J. Med. Econ..

[49] Kim H.-O. (2025). BTK inhibitors and next-generation BTK-targeted therapeutics for B-cell malignancies. Arch. Pharm. Res..

[50] Schmidt A. (2024). Pirtobrutinib for the treatment of B-cell malignancies: Recent developments. Hematol. Blood Transfus..

[51] Wang M.L., Rule S., Martin P., Goy A., Auer R., Kahl B.S., Jurczak W., Advani R.H., Romaguera J.E., Williams M.E. (2013). Targeting BTK with ibrutinib in relapsed or refractory mantle-cell lymphoma. N. Engl. J. Med..

[52] Burger J.A., Tedeschi A., Barr P.M., Robak T., Owen C., Ghia P., Bairey O., Hillmen P., Bartlett N.L., Li J. (2015). Ibrutinib as initial therapy for patients with chronic lymphocytic leukemia. N. Engl. J. Med..

[53] Ahn I.E., Underbayev C., Albitar A., Herman S.E.M., Tian X., Maric I., Arthur D.C., Wake L., Pittaluga S., Yuan C.M. (2017). Clonal evolution leading to ibrutinib resistance in chronic lymphocytic leukemia. Blood.

[54] Wang M., Rule S., Zinzani P.L., Goy A., Casasnovas O., Smith S.D., Damaj G., Doorduijn J., Lamy T., Morschhauser F. (2018). Acalabrutinib in relapsed or refractory mantle cell lymphoma (ACE-LY-004): A single-arm, multicentre, phase 2 trial. Lancet.

[55] Gomez E.B., Ebata K., Randeria H.S., Rosendahl M.S., Cedervall E.P., Morales T.H., Hanson L.M., Brown N.E., Gong X., Stephens J. (2023). Preclinical characterization of pirtobrutinib, a highly selective, noncovalent (reversible) BTK inhibitor. Blood.

[56] Nakhoda S., Vistarop A., Wang Y.L. (2023). Resistance to Bruton tyrosine kinase inhibition in chronic lymphocytic leukaemia and non-Hodgkin lymphoma. Br. J. Haematol..

[57] Mouhssine S., Maher N., Matti B.F., Alwan A.F., Gaidano G. (2024). Targeting BTK in B cell malignancies: From mode of action to resistance mechanisms. Int. J. Mol. Sci..

[58] Davis R.E., Ngo V.N., Lenz G., Tolar P., Young R.M., Romesser P.B., Kohlhammer H., Lamy L., Zhao H., Yang Y. (2010). Chronic active B-cell-receptor signalling in diffuse large B-cell lymphoma. Nature.

[59] Valla K., Flowers C.R., Koff J.L. (2018). Targeting the B cell receptor pathway in non-Hodgkin lymphoma. Expert Opin. Investig. Drugs.

[60] Sibaud V., Beylot-Barry M., Protin C., Vigarios E., Recher C., Ysebaert L. (2020). Dermatological Toxicities of Bruton’s Tyrosine Kinase Inhibitors. Am. J. Clin. Dermatol..

[61] Robak T., Witkowska M., Smolewski P. (2022). The role of Bruton’s kinase inhibitors in chronic lymphocytic leukemia: Current status and future directions. Cancers.

[62] Gu D., Tang H., Wu J., Li J., Miao Y. (2021). Targeting Bruton tyrosine kinase using non-covalent inhibitors in B cell malignancies. J. Hematol. Oncol..

[63] Shah N.N., Wang M., Roeker L.E., Patel K., Woyach J.A., Wierda W.G., Ujjani C.S., Eyre T.A., Zinzani P.L., Alencar A.J. (2025). Pirtobrutinib monotherapy in Bruton tyrosine kinase inhibitor-intolerant patients with B-cell malignancies: Results of the phase I/II BRUIN trial. Haematologica.

[64] Jensen J.L., Mato A.R., Pena C., Roeker L.E., Coombs C.C. (2022). The potential of pirtobrutinib in multiple B-cell malignancies. Ther. Adv. Hematol..

[65] Keam S.J. (2023). Pirtobrutinib: First approval. Drugs.

[66] Davis D.D., Ohana Z., Pham H.M. (2024). Pirtobrutinib: A novel non-covalent BTK inhibitor for the treatment of adults with relapsed/refractory mantle cell lymphoma. J. Oncol. Pharm. Pract..

[67] De S.K. (2024). Pirtobrutinib: First non-covalent tyrosine kinase inhibitor for treating relapsed or refractory mantle cell lymphoma in adults. Curr. Med. Chem..

[68] Pirtobrutinib is an Orally Active, Highly Selective and non-Covalent BTK Inhibitor. Network of Cancer Research. https://www.cancer-research-network.com/2021/05/06/pirtobrutinib-is-an-orally-active-highly-selective-and-non-covalent-btk-inhibitor/.

[69] Mato A.R., Woyach J.A., Brown J.R., Ghia P., Patel K., Eyre T.A., Munir T., Lech-Maranda E., Lamanna N., Tam C.S. (2023). Pirtobrutinib after a covalent BTK inhibitor in chronic lymphocytic leukemia. N. Engl. J. Med..

[70] Ibrahim S., Pham N., Sahni A., Sekeres S., Cool A., Taylor J., Coombs C.C. (2025). Pirtobrutinib in the treatment of chronic lymphocytic leukemia or small lymphocytic lymphoma. Future Oncol..

[71] Molica S., Allsup D. (2025). Pirtobrutinib in chronic lymphocytic leukemia: Navigating resistance and the personalisation of BTK-targeted therapy. Cancers.

[72] Wang M.L., Jurczak W., Zinzani P.L., Eyre T.A., Cheah C.Y., Ujjani C.S., Koh Y., Izutsu K., Gerson J.N., Flinn I.W. (2023). Pirtobrutinib in covalent Bruton tyrosine kinase inhibitor pretreated mantle-cell lymphoma. J. Clin. Oncol..

[73] Aydilek E., Wulf G., Schwarz F., Bacher U., Rummel M., Stiefel O., Kerkhoff A., Maulhardt M., Melchardt T., Pabst T. (2024). Outcomes of pirtobrutinib for relapsed/refractory mantle cell lymphoma in a compassionate use program in Europe. Cancer Med..

[74] Shah N.N., Zinzani P.L., Wang M., Nasta S.D., Lech-Maranda E., Ogawa Y., Fakhri B., Kuss B., Miyashita K., Patel K. (2025). Pirtobrutinib, a highly selective, noncovalent (reversible) BTKi in relapsed/refractory follicular lymphoma: Phase 1/2 BRUIN study. Blood Adv..

[75] Patel K., Vose J.M., Nasta S.D., Brown J.R., Maddocks K.J., Woyach J.A., Shah N.N., Fakhri B., Tessoulin B., Ma S. (2026). Pirtobrutinib, a highly selective, noncovalent (reversible) BTKi in relapsed/refractory marginal zone lymphoma: Phase 1/2 BRUIN study. Blood Adv..

[76] Thompson P.A., Siddiqi T. (2022). Treatment of Richter’s syndrome. Hematol. Am. Soc. Hematol. Educ. Program.

[77] Le H., Baek G., Huang I., Siu C., Shadman M. (2025). The evolving therapeutic landscape of Richter transformation. Curr. Hematol. Malig. Rep..

[78] Eyre T.A., Shah N.N., Dreyling M., Jurczak W., Wang Y., Cheah C.Y., Song Y., Gandhi M., Chay C., Sharman J. (2022). BRUIN MCL-321: Phase III study of pirtobrutinib versus investigator choice of BTK inhibitor in BTK inhibitor-naive mantle cell lymphoma. Future Oncol..

[79] Salles G., Chen J.M.H., Zhang I., Kerbauy F., Wu J.J., Wade S.W., Nunes A., Feng C., Kloos I., Peng W. (2024). Matching-adjusted indirect comparison of brexucabtagene autoleucel (ZUMA-2) and pirtobrutinib (BRUIN) in patients with relapsed/refractory mantle cell lymphoma previously treated with a covalent Bruton tyrosine kinase inhibitor. Adv. Ther..

[80] Yoon J.T., Zhou Y., Mikhaleva M., Choi D.S., Fernandes S.M., Armand P., Bessnow A.C., Crombie J.L., Fisher D.C., Jacobsen E.D. (2025). Characteristics and outcomes of patients with double refractory and double exposed chronic lymphocytic leukemia. Blood Adv..

[81] Islam P. (2023). Current treatment options in relapsed and refractory chronic lymphocytic leukemia/small lymphocytic lymphoma: A review. Curr. Treat. Options Oncol..

[82] Sharman J.P., Jurczak W., Coombs C.C., Hill M., Wang D., Ku N.C., Guntur A., Shahda S., Leow C.C., Ghia P. (2022). PB1864: BRUIN CLL-321: A phase 3 open-label, randomized study of pirtobrutinib vs investigator’s choice of idelalisib + rituximab or bendamustine + rituximab in BTKi pretreated CLL/SLL (trial in progress). Hemasphere.

[83] Tantawy S.I., Aslan B., Manyam G., Iles L.R., Timofeeva N., Singh N., Jain N., Ferrajoli A., Thompson P.A., Patel K.P. (2025). Pharmacological profiling in CLL patients during pirtobrutinib therapy and disease progression. Blood Cancer J..

[84] Woyach J.A., Qiu L., Grosicki S., Wrobel T., Capra M., Czyz J., Yi S., Eom K.-S., Panovská A., Jurczak W. (2026). Pirtobrutinib versus ibrutinib in treatment-naïve and relapsed/refractory chronic lymphocytic leukemia/small lymphocytic lymphoma. J. Clin. Oncol..

[85] Xu J., Lin J., Gan H., He Q., Wang W., Liu Y. (2025). Investigation of the mechanism of hypertension caused by BTKi in the treatment of hematologic diseases. Front. Pharmacol..

[86] Quartermaine C., Ghazi S.M., Yasin A., Awan F.T., Fradley M., Wiczer T., Kalathoor S., Ferdousi M., Krishan S., Habib A. (2023). Cardiovascular toxicities of BTK inhibitors in chronic lymphocytic leukemia: JACC: CardioOncology state-of-the-art review. JACC CardioOncol..

[87] Gambril J.A., Ghazi S.M., Sansoterra S., Ferdousi M., Kola-Kehinde O., Ruz P., Kittai A.S., Rogers K., Grever M., Bhat S. (2024). Atrial fibrillation burden and clinical outcomes following BTK inhibitor initiation. Leukemia.

[88] Shadman M. (2023). Diagnosis and treatment of chronic lymphocytic leukemia: A review. JAMA.

[89] Yi S., Cao J., Feng R., Zhou K., Qiu L., Li F., Yang H.Y., Zhou H., Hui W., Cui J. (2026). A Comprehensive Analysis of Pirtobrutinib in Chinese Patients with Chronic Lymphocytic Leukaemia/Small Lymphocytic Lymphoma (CLL/SLL): Results from the Phase 3 Study BRUIN CLL-321. Br. J. Haematol..

[90] Mehra S., Nicholls M., Taylor J. (2024). The evolving role of Bruton’s tyrosine kinase inhibitors in B cell lymphomas. Int. J. Mol. Sci..

[91] Kojima K., Watanabe S., Takeuchi A., Kunisawa N., Yoshida S. (2025). BTK inhibitors potently impair platelet aggregation as a class effect independent of BTK specificity or dose in CLL and MCL. J. Clin. Exp. Hematop..

[92] Dasanu C.A., Mann S.K., Baidya M., Mdluli X.P., Stapleton A.E., Codreanu I. (2024). Evaluation of infectious morbidity due to BTK inhibitors in indolent B-cell lymphomas: Latest research findings and systematic analysis. Expert Opin. Pharmacother..

[93] Coombs C.C., Woyach J.A., Brown J.R., Ghia P., Roeker L.E., Patel K., Eyre T.A., Tam C.S., Seymour J.F., Shah N.N. (2025). Patient-reported outcomes among patients with mantle cell lymphoma or chronic lymphocytic leukemia receiving pirtobrutinib in the BRUIN phase 1/2 study: Final analysis. Curr. Med. Res. Opin..

[94] Cappell K.M., Kochenderfer J.N. (2023). Long-term outcomes following CAR T cell therapy: What we know so far. Nat. Rev. Clin. Oncol..

[95] Study Evaluating Pirtobrutinib in Combination with Rituximab and Venetoclax in Participants with Previously Untreated Mantle Cell Lymphoma (GATE1). Identifier: NCT06522386. ClinicalTrials.gov. NCT06522386.

[96] Study of Pirtobrutinib in Combination with Venetoclax in Participants with Relapsed/Refractory Mantle Cell Lymphoma. Identifier: NCT05529069. ClinicalTrials.gov. NCT05529069.

[97] Study of Pirtobrutinib in Combination with Rituximab in Participants with Previously Untreated Mantle Cell Lymphoma. Identifier: NCT06263491. ClinicalTrials.gov. NCT06263491.

[98] Study of Pirtobrutinib in Combination with Glofitamab in Participants with Relapsed/Refractory Mantle Cell Lymphoma. Identifier: NCT06252675. ClinicalTrials.gov. NCT06252675.

[99] Wang W., Cai Q., Liu Y., Nie L., Lee H.H., Yan F., Fei Y., Yao Y., Li Y., Tan L. (2025). TCA cycle mode switch determines the fate of pirtobrutinib-tolerant persister cells in mantle cell lymphoma. Blood.

[100] Zhang J., Lu X., Li J., Miao Y. (2022). Combining BTK inhibitors with BCL2 inhibitors for treating chronic lymphocytic leukemia and mantle cell lymphoma. Biomark. Res..

[101] Sagiv-Barfi I., Kohrt H.E.K., Czerwinski D.K., Ng P.P., Chang B.Y., Levy R. (2015). Therapeutic antitumor immunity by checkpoint blockade is enhanced by ibrutinib, an inhibitor of both BTK and ITK. Proc. Natl. Acad. Sci. USA.

[102] Jiang Q., Peng Y., Herling C.D., Herling M. (2024). The immunomodulatory mechanisms of BTK inhibition in CLL and beyond. Cancers.

[103] Wang E., Mi X., Thompson M.C., Montoya S., Notti R.Q., Afaghani J., Durham B.H., Penson A., Witkowski M.T., Lu S.X. (2022). Mechanisms of resistance to noncovalent Bruton’s tyrosine kinase inhibitors. N. Engl. J. Med..

[104] Wiśniewski K., Puła B. (2024). A review of resistance mechanisms to Bruton’s kinase inhibitors in chronic lymphocytic leukemia. Int. J. Mol. Sci..

[105] Burger J.A., Landau D.A., Taylor-Weiner A., Bozic I., Zhang H., Sarosiek K., Wang L., Stewart C., Fan J., Hoellenriegel J. (2016). Clonal evolution in patients with chronic lymphocytic leukaemia developing resistance to BTK inhibition. Nat. Commun..

[106] Molica S. (2023). Redefining efficacy and safety endpoints for chronic lymphocytic leukemia in the era of targeted therapy. Expert Rev. Hematol..

[107] Montoya S., Thompson M.C. (2023). Non-covalent Bruton’s tyrosine kinase inhibitors in the treatment of chronic lymphocytic leukemia. Cancers.

[108] Tam C.S., Thompson P.A. (2024). BTK inhibitors in CLL: Second-generation drugs and beyond. Blood Adv..

[109] Molica S., Allsup D. (2026). Pirtobrutinib at the crossroads: Shaping its future role in chronic lymphocytic leukemia (CLL) care. Eur. J. Haematol..

[110] JAYPIRCA (pirtobrutinib) Prescribing Information. https://pi.lilly.com/us/jaypirca-uspi.pdf.

[111] Wang J.F., Wang Y. (2024). Evaluating pirtobrutinib for the treatment of relapsed or refractory mantle cell lymphoma. Expert Rev. Hematol..

[112] Wierda W.G., Shah N.N., Cheah C.Y., Lewis D., Hoffmann M.S., Coombs C.C., Lamanna N., Ma S., Jagadeesh D., Munir T. (2024). Pirtobrutinib, a highly selective, non-covalent (reversible) BTK inhibitor in patients with B-cell malignancies: Analysis of the Richter transformation subgroup from the multicentre, open-label, phase 1/2 BRUIN study. Lancet Haematol..

[113] Younes A., Bartlett N.L., Leonard J.P., Kennedy D.A., Lynch C.M., Sievers E.L., Forero-Torres A. (2010). Brentuximab vedotin (SGN-35) for relapsed CD30-positive lymphomas. N. Engl. J. Med..

[114] Senter P.D., Sievers E.L. (2012). The discovery and development of brentuximab vedotin for use in relapsed Hodgkin lymphoma and systemic anaplastic large cell lymphoma. Nat. Biotechnol..

[115] Stein H., Falini B. (2025). CD30 as a target molecule in the diagnosis and therapy of lymphomas. Am. J. Hematol..

[116] van der Weyden C.A., Pileri S.A., Feldman A.L., Whisstock J., Prince H.M. (2017). Understanding CD30 biology and therapeutic targeting: A historical perspective providing insight into future directions. Blood Cancer J..

[117] Ansell S.M., Radford J., Connors J.M., Długosz-Danecka M., Kim W.-S., Gallamini A., Ramchandren R., Friedberg J.W., Advani R., Hutchings M. (2022). Overall survival with brentuximab vedotin in stage III or IV Hodgkin’s lymphoma. N. Engl. J. Med..

[118] Tanaka Y. (2025). Prognostic and immunomodulatory roles of CD30 in diffuse large B-cell lymphoma: A cell-of-origin- and microenvironment-dependent paradigm. J. Clin. Exp. Hematop..

[119] van de Donk N.W.C.J., Dhimolea E. (2012). Brentuximab vedotin. MAbs.

[120] Chen J., Jaracz S., Zhao X., Chen S., Ojima I. (2005). Antibody-Cytotoxic Agent Conjugates for Cancer Therapy. Expert Opin. Drug Deliv..

[121] Schirrmann T., Steinwand M., Wezler X., Ten Haaf A., Tur M.K., Barth S. (2014). CD30 as a Therapeutic Target for Lymphoma. BioDrugs.

[122] Wang Y., Cheng X., Li X., Chen W., Zhang X., Liu Y. (2025). Bystander effect in antibody–drug conjugates: Navigating the fine line in tumor heterogeneity. Crit. Rev. Oncol. Hematol..

[123] Shi R., Jia L., Lv Z., Cui J. (2025). Another power of antibody–drug conjugates: Immunomodulatory effect and clinical applications. Front. Immunol..

[124] Collins G.P., Bruce D., Eyre T.A. (2014). New therapies in T-cell lymphoma. Lymphoma Chronic Lymphocytic Leuk..

[125] Younes A., Gopal A.K., Smith S.E., Ansell S.M., Rosenblatt J.D., Savage K.J., Ramchandren R., Bartlett N.L., Cheson B.D., de Vos S. (2012). Results of a pivotal phase II study of brentuximab vedotin for patients with relapsed or refractory Hodgkin’s lymphoma. J. Clin. Oncol..

[126] Bradley A.M., Devine M., DeRemer D. (2013). Brentuximab vedotin: An anti-CD30 antibody–drug conjugate. Am. J. Health Syst. Pharm..

[127] Ogura M., Tobinai K., Hatake K., Ishizawa K., Uike N., Uchida T., Suzuki T., Aoki T., Watanabe T., Maruyama D. (2014). Phase I/II study of brentuximab vedotin in Japanese patients with relapsed or refractory CD30-positive Hodgkin’s lymphoma or systemic anaplastic large-cell lymphoma. Cancer Sci..

[128] Chen R., Chen B. (2015). Brentuximab vedotin for relapsed or refractory Hodgkin’s lymphoma. Drug Des. Devel. Ther..

[129] Scott L.J. (2017). Brentuximab vedotin: A review in CD30-positive Hodgkin lymphoma. Drugs.

[130] Horwitz S., O’Connor O.A., Pro B., Trümper L., Iyer S., Advani R., Bartlett N.L., Christensen J.H., Morschhauser F., Domingo-Domenech E. (2022). The ECHELON-2 trial: 5-year results of a randomized, phase III study of brentuximab vedotin with chemotherapy for CD30-positive peripheral T-cell lymphoma. Ann. Oncol..

[131] Akkad N., Mehta-Shah N. (2026). SOHO state of the art updates and next questions: Optimizing frontline treatment for peripheral T-cell lymphoma. Clin. Lymphoma Myeloma Leuk..

[132] Saleh N., O’Connor O.A. (2026). Are CHOP-plus really CHOP-minus propositions in the treatment of PTCL? A comprehensive assessment of the strategy. Blood Adv..

[133] Kim T.Y., Kim T.J., Han E.J., Min G.J., Cho S.G., Jeon Y. (2025). Impact of KMT2A rearrangement on peripheral T-cell lymphoma, not otherwise specified, and angioimmunoblastic T-cell lymphoma. Biomedicines.

[134] Sidaway P. (2018). Haematological Cancer: Brentuximab Effective in Untreated Hodgkin Lymphoma. Nat. Rev. Clin. Oncol..

[135] Picardi M., Vincenzi A., Giordano C., Pugliese N., Scarpa A., Lombardi A., Vigliar E., Troncone G., Cappiello R., Mascolo M. (2026). Brentuximab vedotin with adriamycin, vinblastine, and dacarbazine for patients aged 18–59 years with untreated advanced stage classical Hodgkin lymphoma: The largest real-life series from Southern Italy cancer centers. Eur. J. Haematol..

[136] Herrera A.F., LeBlanc M., Castellino S.M., Li H., Rutherford S.C., Evens A.M., Davison K., Punnett A., Parsons S.K., Ahmed S. (2024). Nivolumab Plus AVD in Advanced-Stage Classic Hodgkin’s Lymphoma. N. Engl. J. Med..

[137] Moskowitz C.H., Nademanee A., Masszi T., Agura E., Holowiecki J., Abidi M.H., Chen A.I., Stiff P., Gianni A.M., Carella A. (2015). Erratum in: Department of Error: Brentuximab vedotin as consolidation therapy after autologous stem-cell transplantation in patients with Hodgkin’s lymphoma at risk of relapse or progression (AETHERA): A randomised, double-blind, placebo-controlled, phase 3 trial. Lancet.

[138] Moskowitz C.H., Walewski J., Nademanee A., Masszi T., Agura E., Holowiecki J., Abidi M.H., Chen A.I., Stiff P., Viviani S. (2018). Five-year PFS from the AETHERA trial of brentuximab vedotin for Hodgkin lymphoma at high risk of progression or relapse. Blood.

[139] Koyun D., Şahin U., Cengiz Seval G., Gören D., Gökmen A., Civriz Bozdağ S., Toprak S.K., Kurt Yüksel M., Topçuoğlu P., Arslan Ö. (2026). Allogeneic hematopoietic cell transplantation for relapsed/refractory Hodgkin lymphoma: A multicenter real-world experience. Turk. J. Haematol..

[140] Prince H.M., Kim Y.H., Horwitz S.M., Dummer R., Scarisbrick J., Quaglino P., Zinzani P.L., Wolter P., Sanches J.A., Ortiz-Romero P.L. (2017). Brentuximab vedotin or physician’s choice in CD30-positive cutaneous T-cell lymphoma (ALCANZA): An international, open-label, randomised, phase 3, multicentre trial. Lancet.

[141] Dummer R., Prince H.M., Whittaker S., Horwitz S.M., Kim Y.H., Scarisbrick J., Quaglino P., Zinzani P.L., Wolter P., Eradat H. (2020). Patient-Reported Quality of Life in Patients with Relapsed/Refractory Cutaneous T-Cell Lymphoma: Results from the Randomised Phase III ALCANZA Study. Eur. J. Cancer.

[142] Bartlett N.L., Hahn U., Kim W.-S., Fleury I., Laribi K., Bergua J.-M., Bouabdallah K., Forward N., Bijou F., MacDonald D. (2025). Brentuximab vedotin combination for relapsed diffuse large B-cell lymphoma. J. Clin. Oncol..

[143] Bartlett N.L., Hahn U., Kim W.-S., Fleury I., Laribi K., Bergua J.-M., Bouabdallah K., Forward N., Bijou F., MacDonald D. (2025). Plain language summary of the ECHELON-3 study: Brentuximab vedotin combination treatment in people with diffuse large B-cell lymphoma. Future Oncol..

[144] Chong E.A., Tomasulo E.B., Barta S.K. (2026). 2026 update on the management of diffuse large B-cell lymphoma. Am. J. Hematol..

[145] Herrera A.F., Chen L., Nieto Y., Holmberg L., Johnston P., Mei M., Popplewell L., Armenian S., Cao T., Farol L. (2023). Brentuximab vedotin plus nivolumab after autologous haematopoietic stem-cell transplantation for adult patients with high-risk classic Hodgkin lymphoma: A multicentre, phase 2 trial. Lancet Haematol..

[146] Massaro F., Meuleman N., Bron D., Vercruyssen M., Maerevoet M. (2022). Brentuximab vedotin and pembrolizumab combination in patients with relapsed/refractory Hodgkin lymphoma: A single-centre retrospective analysis. Cancers.

[147] Chen R.W. (2018). Is There a Place for the Combination of Brentuximab Vedotin and Bendamustine in Treatment of Patients with Relapsed/Refractory Hodgkin Lymphoma?. Ann. Transl. Med..

[148] Tournilhac O., Bouabdallah K., Lecolant S., Hacini M., Laribi K., Bailly S., Belmondo T., Maerevoet M., Ysebaert L., Guidez S. (2025). Brentuximab vedotin addition to gemcitabine in relapsed or refractory peripheral T-cell lymphoma: A LYSA phase 2 study. Blood Adv..

[149] Makita S., Maruyama D., Tobinai K. (2020). Safety and efficacy of brentuximab vedotin in the treatment of classic Hodgkin lymphoma. Onco Targets Ther..

[150] Yi J.H., Park Y.H., Lee M.W., Yoo K.H., Hong J., Eom H.S. (2025). Brentuximab vedotin combined with cisplatin, cytarabine, and dexamethasone treatment in transplant-eligible Korean patients with relapsed or refractory Hodgkin’s lymphoma. Blood Res..

[151] Rausch C., Bacher U., Rabaglio M., Vorburger C., Klingenberg A., Banz Y., Daskalakis M., Pabst T. (2025). Randomized phase II study of brentuximab vedotin with high-dose chemotherapy in CD30-positive lymphoma. Hematol. Oncol..

[152] Abramson J.S., Stuver R., Herrera A., Patterson E., Wen Y.P., Moskowitz A. (2024). Management of peripheral neuropathy associated with brentuximab vedotin in the frontline treatment of classical Hodgkin lymphoma. Crit. Rev. Oncol. Hematol..

[153] Advani R.H., Moskowitz A.J., Bartlett N.L., Vose J.M., Ramchandren R., Feldman T.A., LaCasce A.S., Christian B.A., Ansell S.M., Moskowitz C.H. (2021). Brentuximab vedotin in combination with nivolumab in relapsed or refractory Hodgkin lymphoma: 3-year study results. Blood.

[154] Vassilakopoulos T.P. (2021). Relapsed or Refractory Classical Hodgkin Lymphoma: Which Immunotherapy, and When?. Lancet Oncol..

[155] Johnson N.A., Zinzani P.L., Domingo-Domenech E., Brody J., Kline J., Shah B.D., Mehta A.N., Ghesquieres H., Savage K.J., Barr P.M. (2026). Nivolumab plus brentuximab vedotin for relapsed/refractory diffuse large B-cell lymphoma. Future Oncol..

[156] Carson K.R., Newsome S.D., Kim E.J., Wagner-Johnston N.D., von Geldern G., Moskowitz C.H., Moskowitz A.J., Rook A.H., Jalan P., Loren A.W. (2014). Progressive multifocal leukoencephalopathy associated with brentuximab vedotin therapy: A report of 5 cases from the Southern Network on Adverse Reactions (SONAR) project. Cancer.

[157] Jagadeesh D., Horwitz S., Bartlett N.L., Kim Y., Jacobsen E., Duvic M., Little M., Trepicchio W., Fenton K., Onsum M. (2022). Response to brentuximab vedotin by CD30 expression in non-Hodgkin lymphoma. Oncologist.

[158] Dumitru A.V., Țăpoi D.A., Halcu G., Munteanu O., Dumitrascu D.-I., Ceaușu M.C., Gheorghișan-Gălățeanu A.-A. (2023). The polyvalent role of CD30 for cancer diagnosis and treatment. Cells.

[159] Gu A., Masturov Y., Rekant M., Miller A. (2026). Brentuximab-induced peripheral neuropathy in the setting of radiation-induced brachial plexopathy. Cureus.

[160] Reagan P.M., Magnuson A., Portell C.A., Baran A., Casulo C., Williams A.R., Wallace D.S., Barr P.M., Friedberg J.W. (2026). Brentuximab vedotin and dose-attenuated chemoimmunotherapy for patients 75 years and older with diffuse large B-cell lymphoma with analysis of outcomes by frailty. J. Geriatr. Oncol..

[161] Yasuda H., Yasuda M., Komatsu N. (2021). Chemotherapy for non-Hodgkin lymphoma in the hemodialysis patient: A comprehensive review. Cancer Sci..

[162] Stuver R., Epstein-Peterson Z.D., Johnson W.T., Khan N., Lewis N., Moskowitz A.J., Sauter C.S., Horwitz S. (2022). Current treatment of peripheral T-cell lymphoma. Oncology.

[163] Degtyarev E., Bolaños N., Brody J.D., Buchbinder A., Buyse M., Fuchs M., Halabi S., Hemmings R., Masood A., Newsome S. (2024). End points in clinical trials in diffuse large B-cell lymphoma: Time for more dialogue?. Future Oncol..

[164] Berger G.K., McBride A., Lawson S., Royball K., Yun S., Gee K., Riaz I.B., Saleh A.A., Puvvada S., Anwer F. (2016). Brentuximab vedotin for treatment of non-Hodgkin lymphomas: A systematic review. Crit. Rev. Oncol. Hematol..

[165] Yang Y., Liu S.-R., Wang L., Zhang R., Xie J. (2025). Frontline brentuximab vedotin-based therapy for newly diagnosed classical Hodgkin lymphoma: A meta-analysis of randomized controlled trials. Front. Oncol..

[166] Schroers-Martin J.G., Advani R. (2024). When should we use it? The role of brentuximab vedotin in 2024. Hematol. Am. Soc. Hematol. Educ. Program.

[167] Delacruz W., Setlik R., Hassantoufighi A., Daya S., Cooper S., Selby D., Brown A. (2016). Novel brentuximab vedotin combination therapies show promising activity in highly refractory CD30+ non-Hodgkin lymphoma: A case series and review of the literature. Case Rep. Oncol. Med..

[168] Sureda A., Pavlovsky A., Haidar D., Kristo F., Stache V., Zomas A. (2025). Real-world outcomes of brentuximab vedotin as consolidation therapy after autologous stem cell transplantation in relapsed/refractory Hodgkin lymphoma: A systematic review and meta-analysis. Bone Marrow Transpl..

[169] Shah G.L., Moskowitz C.H. (2018). Transplant strategies in relapsed/refractory Hodgkin lymphoma. Blood.

[170] Massa H., Massaro F., Maerevoet M. (2025). Combination of brentuximab vedotin and pembrolizumab as salvage treatment before autologous stem cell transplantation and maintenance in patients with relapsed/refractory Hodgkin lymphoma. Biomedicines.

[171] He L., Chen N., Dai L., Peng X. (2023). Advances and challenges of immunotherapies in NK/T cell lymphomas. iScience.

[172] Zhang H., Yan Y., Yi S., Sun Q. (2025). Advancements in targeting CD30 for lymphoma therapy: A historical perspective and future directions. Expert Rev. Hematol..

[173] Zhang Q., Liu Y. (2026). Survival trends and prognostic modeling in ALK-positive anaplastic large cell lymphoma: A population-based study in the brentuximab vedotin era. Cancer Med..

[174] ADCETRIS (Brentuximab Vedotin) Prescribing Information. Seagen Inc.. https://labeling.pfizer.com/ShowLabeling.aspx?id=20629.

[175] Brentuximab Vedotin (Adcetris) Prescribing Information. U.S. Food and Drug Administration. https://www.accessdata.fda.gov/drugsatfda_docs/label/2014/125388_s056s078lbl.pdf.

[176] Prince H.M., Dickinson M. (2012). Romidepsin for cutaneous T-cell lymphoma. Clin. Cancer Res..

[177] Lee H.-Z., Kwitkowski V.E., Del Valle P.L., Ricci M.S., Saber H., Habtemariam B.A., Bullock J., Bloomquist E., Shen Y.L., Chen X.-H. (2015). FDA Approval: Belinostat for the Treatment of Patients with Relapsed or Refractory Peripheral T-cell Lymphoma. Clin. Cancer Res..

[178] Dawson M.A., Kouzarides T. (2012). Cancer epigenetics: From mechanism to therapy. Cell.

[179] Jones P.A., Baylin S.B. (2007). The epigenomics of cancer. Cell.

[180] Iqbal J., Wright G., Wang C., Rosenwald A., Gascoyne R.D., Weisenburger D.D., Greiner T.C., Smith L., Guo S., Wilcox R.A. (2014). Gene expression signatures delineate biological and prognostic subgroups in peripheral T-cell lymphoma. Blood.

[181] Marks P.A., Xu W.-S. (2009). Histone deacetylase inhibitors: Potential in cancer therapy. J. Cell Biochem..

[182] West A.C., Johnstone R.W. (2014). New and emerging HDAC inhibitors for cancer treatment. J. Clin. Investig..

[183] Patel R., Modi A., Vekariya H. (2024). Discovery and development of HDAC inhibitors: Approaches for the treatment of cancer—A mini-review. Curr. Drug Discov. Technol..

[184] Zhang Q., Wang S., Chen J., Yu Z. (2019). Histone deacetylases (HDACs) guided novel therapies for T-cell lymphomas. Int. J. Med. Sci..

[185] Li Y., Wang F., Chen X., Wang J., Zhao Y., Li Y., He B. (2019). Zinc-dependent deacetylase (HDAC) inhibitors with different zinc binding groups. Curr. Top. Med. Chem..

[186] Bolden J.E., Peart M.J., Johnstone R.W. (2006). Anticancer activities of histone deacetylase inhibitors. Nat. Rev. Drug Discov..

[187] Kroesen M., Gielen P., Brok I.C., Armandari I., Hoogerbrugge P.M., Adema G.J. (2014). HDAC inhibitors and immunotherapy: A double-edged sword?. Oncotarget.

[188] Apuri S., Sokol L. (2016). An overview of investigational histone deacetylase inhibitors (HDACis) for the treatment of non-Hodgkin’s lymphoma. Expert Opin. Investig. Drugs.

[189] Chung C. (2019). Current targeted therapies in lymphomas. Am. J. Health Syst. Pharm..

[190] Lu G., Jin S., Lin S., Gong Y., Zhang L., Yang J., Mou W., Du J. (2023). Update on histone deacetylase inhibitors in peripheral T-cell lymphoma (PTCL). Clin. Epigenet..

[191] Beleodaq^®^ (Belinostat) for Injection. https://beleodaq.com/hcp/about.

[192] Moskowitz A.J., Horwitz S.M. (2017). Targeting histone deacetylases in T-cell lymphoma. Leuk. Lymphoma.

[193] Poole R.M. (2014). Belinostat: First global approval. Drugs.

[194] Thompson C.A. (2014). Belinostat approved for use in treating rare lymphoma. Am. J. Health Syst. Pharm..

[195] Rashidi A., Cashen A.F. (2015). Belinostat for the treatment of relapsed or refractory peripheral T-cell lymphoma. Future Oncol..

[196] Cliff E.R.S., Russler-Germain D.A., Daval C.J.R., Kesselheim A.S. (2024). US Food and Drug Administration’s directive to deal with delayed confirmatory trials: Lessons from pralatrexate and belinostat for T-cell lymphoma. J. Clin. Oncol..

[197] Vose J., Armitage J., Weisenburger D., International T-Cell Lymphoma Project (2008). International peripheral T-cell and natural killer/T-cell lymphoma study: Pathology findings and clinical outcomes. J. Clin. Oncol..

[198] Broccoli A., Zinzani P.L. (2017). Peripheral T-cell lymphoma, not otherwise specified. Blood.

[199] Gisselbrecht C., Sibon D. (2018). New perspectives in the therapeutic approach of peripheral T-cell lymphoma. Curr. Opin. Oncol..

[200] Ito Y., Makita S., Tobinai K. (2019). Development of new agents for peripheral T-cell lymphoma. Expert Opin. Biol. Ther..

[201] O’Connor O.A., Ma H., Chan J.Y.S., Kim S.J., Yoon S.E., Kim W.S. (2024). Peripheral T-cell lymphoma: From biology to practice to the future. Cancer Treat. Rev..

[202] Steele N.L., Plumb J.A., Vidal L., Tjørnelund J., Knoblauch P., Rasmussen A., Ooi C.E., Buhl-Jensen P., Brown R., Evans T.R.J. (2008). A phase 1 phar-macokinetic and pharmacodynamic study of the histone deacetylase inhibi-tor belinostat in patients with advanced solid tumors. Clin. Cancer Res..

[203] Sawas A., Ma H., Shustov A., Hsu P., Bhat G., Acosta M., Horwitz S., O’Connor O.A. (2020). Characterizing the belinostat response in patients with relapsed or refractory angioimmunoblastic T-cell lymphoma. Leuk. Lymphoma.

[204] Johnston P.B., Cashen A.F., Nikolinakos P.G., Beaven A.W., Barta S.K., Bhat G., Hasal S.J., De Vos S., Oki Y., Deng C. (2021). Belinostat in combination with standard cyclophosphamide, doxorubicin, vincristine and prednisone as first-line treatment for patients with newly diagnosed peripheral T-cell lymphoma. Exp. Hematol. Oncol..

[205] Diyabalanage H.V.K., Granda M.L., Hooker J.M. (2012). Combination therapy: Histone deacetylase inhibitors and platinum-based chemotherapeutics for cancer. Cancer Lett..

[206] Thurn K.T., Thomas S., Moore A., Munster P.N. (2011). Rational therapeutic combinations with histone deacetylase inhibitors for the treatment of cancer. Future Oncol..

[207] Reimer P. (2015). New developments in the treatment of peripheral T-cell lymphoma—Role of belinostat. Cancer Manag. Res..

[208] Odenike O., Halpern A., Godley L.A., Madzo J., Karrison T., Green M., Fulton N., Mattison R.J., Yee K.W.L., Bennett M. (2015). A phase I and pharmacodynamic study of the histone deacetylase inhibitor belinostat plus azacitidine in advanced myeloid neoplasia. Investig. New Drugs.

[209] Horwitz S.M. (2011). The emerging role of histone deacetylase inhibitors in treating T-cell lymphomas. Curr. Hematol. Malig. Rep..

[210] Wang M., Fang X., Wang X. (2020). Emerging role of histone deacetylase inhibitors in the treatment of diffuse large B-cell lymphoma. Leuk. Lymphoma.

[211] Irimia R., Piccaluga P.P. (2024). Histone deacetylase inhibitors for peripheral T-cell lymphomas. Cancers.

[212] Passero F.C., Ravi D., McDonald J.T., Beheshti A., David K.A., Evens A.M. (2020). Combinatorial ixazomib and belinostat therapy induces NFE2L2-dependent apoptosis in Hodgkin and T-cell lymphoma. Br. J. Haematol..

[213] El Omari N., Bakrim S., Khalid A., Albratty M., Abdalla A.N., Lee L.-H., Goh K.W., Ming L.C., Bouyahya A. (2023). Anticancer Clinical Efficiency and Stochastic Mechanisms of Belinostat. Biomed. Pharmacother..

[214] Jimura N., Fujii K., Qiao Z., Tsuchiya R., Yoshimatsu Y., Kondo T., Kanekura T. (2021). Kinome profiling analysis identified Src pathway as a novel therapeutic target in combination with histone deacetylase inhibitors for cutaneous T-cell lymphoma. J. Dermatol. Sci..

[215] Hu X., Li L., Nkwocha J., Vasiyani H., Kazi A., Sebti S., Grant S. (2025). Geranylgeranyl transferase-1 inhibitor GGTI-2417 enhances the anti-tumour efficacy of histone deacetylase inhibitors in different T-cell lymphoma lines. Br. J. Haematol..

[216] Allen P.B., Lechowicz M.J. (2018). Hematologic toxicity is rare in relapsed patients treated with belinostat: A systematic review of belinostat toxicity and safety in peripheral T-cell lymphomas. Cancer Manag. Res..

[217] Begum R., Parsons J.L., Jones A.M. (2025). Adverse drug reaction profiles of histone deacetylase inhibitors. Sci. Rep..

[218] Subramanian S., Bates S.E., Wright J.J., Espinoza-Delgado I., Piekarz R.L. (2010). Clinical toxicities of histone deacetylase inhibitors. Pharmaceuticals.

[219] Wolska-Washer A., Smolewski P., Robak T. (2021). Advances in the pharmacotherapeutic options for primary nodal peripheral T-cell lymphoma. Expert Opin. Pharmacother..

[220] Molife R., Fong P., Scurr M., Judson I., Kaye S., de Bono J. (2007). HDAC inhibitors and cardiac safety. Clin. Cancer Res..

[221] Zhang L.-Y., Wang Y.-Y., Wen R., Zhang T.-N., Yang N. (2025). Role of histone deacetylase and inhibitors in cardiovascular diseases. Cell Prolif..

[222] Steele N.L., Plumb J.A., Vidal L., Tjørnelund J., Knoblauch P., Buhl-Jensen P., Molife R., Brown R., de Bono J.S., Evans T.R.J. (2011). Pharmacokinetic and pharmacodynamic properties of an oral formulation of the histone deacetylase inhibitor belinostat (PXD101). Cancer Chemother. Pharmacol..

[223] Goey A.K.L., Sissung T.M., Peer C.J., Trepel J.B., Lee M.-J., Tomita Y., Ehrlich S., Bryla C., Balasubramaniam S., Piekarz R. (2016). Effects of UGT1A1 genotype on the pharmacokinetics, pharmacodynamics, and toxicities of belinostat administered by 48-hour continuous infusion in patients with cancer. J. Clin. Pharmacol..

[224] Goey A.K., Figg W.D. (2016). UGT genotyping in belinostat dosing. Pharmacol. Res..

[225] Fardi M., Solali S., Farshdousti Hagh M. (2018). Epigenetic mechanisms as a new approach in cancer treatment: An updated review. Genes Dis..

[226] Lyu G., Li D. (2025). PLEKHG7 expression: A biomarker for prognosis and targeted therapy in diffuse large B-cell lymphoma. Protein Pept. Lett..

[227] Molife L.R., de Bono J.S. (2011). Belinostat: Clinical applications in solid tumors and lymphoma. Expert Opin. Investig. Drugs.

[228] Mak V., Hamm J., Chhanabhai M., Shenkier T., Klasa R., Sehn L.H., Villa D., Gascoyne R.D., Connors J.M., Savage K.J. (2013). Survival of patients with peripheral T-cell lymphoma after first relapse or progression: Spectrum of disease and rare long-term survivors. J. Clin. Oncol..

[229] Atalay F., Yeşilaltay A. (2024). Long-term successful use of belinostat in a patient with relapsed-refractory angioimmunoblastic lymphoma who has previously been heavily treated. J. Cancer Res. Ther..

[230] Simon F., Al-Sawaf O., Seymour J.F., Eichhorst B. (2025). Surrogate endpoints in mature B-cell neoplasms—Meaningful or misleading?. Leukemia.

[231] Ma H., Cheng B., Falchi L., Marchi E., Sawas A., Bhagat G., O’Connor O.A. (2020). Survival benefit in patients with peripheral T-cell lymphomas after treatments with novel therapies and clinical trials. Hematol. Oncol..

[232] Cheng Y.Y., Jin H.C., Chan M.W.Y., Chu W.K., Grusch M. (2018). Epigenetic biomarkers in cancer. Dis. Markers.

[233] Islam S., Espitia C.M., Persky D.O., Carew J.S., Nawrocki S.T. (2020). Resistance to histone deacetylase inhibitors confers hypersensitivity to oncolytic reovirus therapy. Blood Adv..

[234] Daśko M., de Pascual-Teresa B., Ortín I., Ramos A. (2022). HDAC inhibitors: Innovative strategies for their design and applications. Molecules.

[235] Bennett R.L., Licht J.D. (2018). Targeting epigenetics in cancer. Annu. Rev. Pharmacol. Toxicol..

[236] Pichler A.S., Amador C., Fujimoto A., Takeuchi K., de Jong D., Iqbal J., Staber P.B. (2024). Advances in peripheral T-cell lymphomas: Pathogenesis, genetic landscapes and emerging therapeutic targets. Histopathology.

[237] Huang Y.-H., Qiu Y.-R., Zhang Q.-L., Cai M.-C., Yu H., Zhang J.-M., Jiang L., Ji M.-M., Xu P.-P., Wang L. (2024). Genomic and transcriptomic profiling of peripheral T cell lymphoma reveals distinct molecular and microenvironment subtypes. Cell Rep. Med..

[238] Harrop S., Abeyakoon C., Van Der Weyden C., Prince H.M. (2021). Targeted Approaches to T-Cell Lymphoma. J. Pers. Med..

[239] Shirouchi Y., Yamauchi N., Maruyama D. (2026). Recent Advances and Future Perspectives in Frontline Treatment of Peripheral T-Cell Lymphoma. Jpn. J. Clin. Oncol..

[240] Hawash M. (2025). Next-Generation HDAC Inhibitors: Advancing Zinc-Binding Group Design for Enhanced Cancer Therapy. Cells.

[241] Rojek A.E., Smith S.M. (2025). Emerging Immunotherapy Advances for Non-Hodgkin Lymphomas: Engaging T Cells in the Fight. Hematol. Am. Soc. Hematol. Educ. Program.

[242] Lunning M.A., Moskowitz A.J., Horwitz S. (2013). Strategies for relapsed peripheral T-cell lymphoma: The tail that wags the curve. J. Clin. Oncol..

[243] Belinostat (Beleodaq) Prescribing Information. U.S. Food and Drug Administration. https://www.accessdata.fda.gov/drugsatfda_docs/label/2014/206256lbl.pdf.

[244] Li Q., Xia C., Li H., Yan X., Yang F., Cao M., Zhang S., Teng Y., He S., Cao M. (2024). Disparities in 36 Cancers across 185 Countries: Secondary Analysis of Global Cancer Statistics. Front. Med..

[245] Campo E. (2023). The 2022 Classifications of Lymphoid Neoplasms: Keynote. Pathologie.

[246] Alizadeh A.A., Eisen M.B., Davis R.E., Ma C., Lossos I.S., Rosenwald A., Boldrick J.C., Sabet H., Tran T., Yu X. (2000). Distinct types of diffuse large B-cell lymphoma identified by gene expression profiling. Nature.

[247] Schmitz R., Wright G.W., Huang D.W., Johnson C.A., Phelan J.D., Wang J.Q., Roulland S., Kasbekar M., Young R.M., Shaffer A.L. (2018). Genetics and pathogenesis of diffuse large B-cell lymphoma. N. Engl. J. Med..

[248] Shadman M., Davids M.S. (2025). How I treat patients with CLL after prior treatment with a covalent BTK inhibitor and a BCL-2 inhibitor. Blood.

[249] Jamroziak K., Puła B., Walewski J. (2017). Current treatment of chronic lymphocytic leukemia. Curr. Treat. Options Oncol..

[250] Nawaratne V., Sondhi A.K., Abdel-Wahab O., Taylor J. (2024). New means and challenges in the targeting of BTK. Clin. Cancer Res..

[251] Sun R., Li R.C., Li H.Y., Wang N., Xu T.Y., Tian S., Fu D., Li C., Zhang F.Y., Feng Y. (2026). Genetic and transcriptomic profiles identify potential therapeutic targets of concurrent follicular and diffuse large B-cell lymphoma and transformed follicular lymphoma. Hematol. Oncol..

[252] Straus D.J., Długosz-Danecka M., Alekseev S., Illés Á., Picardi M., Lech-Maranda E., Feldman T., Smolewski P., Savage K.J., Bartlett N.L. (2020). Brentuximab vedotin with chemotherapy for stage III/IV classical Hodgkin lymphoma: 3-year update of the ECHELON-1 study. Blood.

[253] Liang J.-H., Hua W., Yin H., Li Y., Zhang X.-Y., Liang J.-H., Zhu L.-Q., Gao R., Wang C.-X., Shao Y. (2025). Clinical implications of ctDNA-based minimal residual disease detection in newly diagnosed peripheral T-cell lymphoma: A single-center cohort study. Cell Commun. Signal..

[254] Jean-Louis G., Cherng H.J. (2025). Measurable residual disease testing during treatment with bispecific antibodies for lymphoma. Cancers.

[255] Schuster S.J., Svoboda J., Chong E.A., Nasta S.D., Mato A.R., Anak Ö., Brogdon J.L., Pruteanu-Malinici I., Bhoj V., Landsburg D. (2017). Chimeric antigen receptor T cells in refractory B-cell lymphomas. N. Engl. J. Med..

[256] Neelapu S.S., Chavez J.C., Sehgal A.R., Epperla N., Ulrickson M.L., Bachy E., Munshi P.N., Casulo C., Maloney D.G., de Vos S. (2025). Five-year follow-up analysis of ZUMA-5: Axicabtagene ciloleucel in relapsed/refractory indolent non-Hodgkin lymphoma. J. Clin. Oncol..

[257] Phillips T.J., Carlo-Stella C., Morschhauser F., Bachy E., Crump M., Trněný M., Bartlett N.L., Zaucha J., Wrobel T., Offner F. (2025). Glofitamab in relapsed/refractory mantle cell lymphoma: Results from a phase I/II study. J. Clin. Oncol..

[258] Sehn L.H., Bartlett N.L., Matasar M.J., Schuster S.J., Assouline S.E., Giri P., Kuruvilla J., Shadman M., Cheah C.Y., Dietrich S. (2025). Long-term 3-year follow-up of mosunetuzumab in relapsed or refractory follicular lymphoma after ≥2 prior therapies. Blood.

[259] Tavarozzi R., Maher N., Catania G., Zacchi G., D’Andrea F., Sofia A., Zanni M., Ladetto M. (2026). Evolving therapeutic strategies in mantle cell lymphoma: Advancements and future directions. Leukemia.

[260] Li J., Zhang L., Yu Z., Bao Z., Li D., Wang L. (2026). The impact of AI on modern oncology from early detection to personalized cancer treatment. npj Precis. Oncol..

[261] Wang W., Zhou H., Tan S., Qin D., Wang S., Xu C., Lei X., Li W., Wang L., Fu S. (2026). Dual targeting of PI3Kδ and PPARα enhances antitumor activity via FoxO1 activation in follicular lymphoma. Cell Death Dis..

[262] Tam C.S., Allan J.N., Siddiqi T., Kipps T.J., Jacobs R., Opat S., Barr P.M., Tedeschi A., Trentin L., Bannerji R. (2022). Fixed-duration ibrutinib plus venetoclax for first-line treatment of CLL: Primary analysis of the CAPTIVATE FD cohort. Blood.

[263] Kumar A., Soumerai J., Abramson J.S., Barnes J.A., Caron P., Chhabra S., Chabowska M., Dogan A., Falchi L., Grieve C. (2025). Zanubrutinib, obinutuzumab, and venetoclax for first-line treatment of mantle cell lymphoma with a TP53 mutation. Blood.

[264] Flinn I.W., Hillmen P., Montillo M., Nagy Z., Illés Á., Etienne G., Delgado J., Kuss B.J., Tam C.S., Gasztonyi Z. (2018). The phase 3 DUO trial: Duvelisib vs ofatumumab in relapsed and refractory CLL/SLL. Blood.

[265] Davids M.S., Roberts A.W., Seymour J.F., Pagel J.M., Kahl B.S., Wierda W.G., Puvvada S., Kipps T.J., Anderson M.A., Salem A.H. (2017). Phase I first-in-human study of venetoclax in patients with relapsed or refractory non-Hodgkin lymphoma. J. Clin. Oncol..

[266] Wierda W.G., Allan J.N., Siddiqi T., Kipps T.J., Opat S., Tedeschi A., Badoux X.C., Kuss B.J., Jackson S., Moreno C. (2021). Ibrutinib plus venetoclax for first-line treatment of chronic lymphocytic leukemia: Primary analysis results from the minimal residual disease cohort of the randomized phase II CAPTIVATE study. J. Clin. Oncol..

[267] Feng X., Guo W., Wang Y., Li J., Zhao Y., Qu L., Yan X., Li J., Guo Q., Young K.H. (2022). The short-term efficacy and safety of brentuximab vedotin plus cyclophosphamide, epirubicin and prednisone in untreated PTCL: A real-world, retrospective study. Adv. Ther..

[268] Lynch R.C. (2024). Toward a Cure for Classical Hodgkin Lymphoma without Chemotherapy. Blood.

[269] Shi Y., Li X. (2025). Challenges of CD30 expression and its impact on targeted treatment responses in non-Hodgkin lymphoma: New perspectives for evaluation and validation. Pathol. Res. Pract..

[270] Burke G.A.A. (2021). Brentuximab Vedotin: Frontline Help in ALCL. Blood.

[271] Campbell P., Thomas C.M. (2017). Belinostat for the treatment of relapsed or refractory peripheral T-cell lymphoma. J. Oncol. Pharm. Pract..

[272] Nosaka K. (2026). Recent progress in T-cell malignancies including adult T-cell leukemia-lymphoma. Int. J. Hematol..

[273] Ge T., Gu X., Jia R., Ge S., Chai P., Zhuang A., Fan X. (2022). Crosstalk between metabolic reprogramming and epigenetics in cancer: Updates on mechanisms and therapeutic opportunities. Cancer Commun..

[274] Wang H., Fu B.-B., Gale R.P., Liang Y. (2021). NK-/T-cell lymphomas. Leukemia.

[275] Nagler A., Perriello V.M., Falini L., Falini B. (2023). How I treat refractory/relapsed diffuse large B-cell lymphomas with CD19-directed chimeric antigen receptor T cells. Br. J. Haematol..

[276] Zhou X., Tu S., Wang C., Huang R., Deng L., Song C., Yue C., He Y., Yang J., Liang Z. (2020). Phase I trial of fourth-generation anti-CD19 chimeric antigen receptor T cells against relapsed or refractory B cell non-Hodgkin lymphomas. Front. Immunol..

[277] Hijazi A., Locke F.L., Scholler N., Mattie M., Filosto S., Bedognetti D., Galon J. (2025). Facts and hopes: CAR T-cell therapy and immune contexture in non-Hodgkin lymphoma. Clin. Cancer Res..

[278] Bachiller M., Barceló-Genestar N., Rodriguez-Garcia A., Alserawan L., Dobaño-López C., Giménez-Alejandre M., Castellsagué J., Colell S., Otero-Mateo M., Antoñana-Vildosola A. (2025). ARI0003: Co-transduced CD19/BCMA dual-targeting CAR-T cells for the treatment of non-Hodgkin lymphoma. Mol. Ther..

[279] Wang C., Guo Y., Han F., Zhang Y., Liu C., Wu Z., Tong C., Yang Q., Lu Y., Zhong Y. (2026). Demethylation-primed tandem CD19/CD20 CAR T cells in relapsed/refractory B-cell lymphoma: A phase I/II trial. Nat. Commun..

[280] Watanabe T. (2025). CD19 CAR-T cell therapy for relapsed or refractory nodal and gastrointestinal follicular lymphoma: Current advances and future perspectives. Curr. Gastroenterol. Rep..

[281] Ruella M., Korell F., Porazzi P., Maus M.V. (2023). Mechanisms of resistance to chimeric antigen receptor-T cells in haematological malignancies. Nat. Rev. Drug Discov..

[282] Tang W., Yu S. (2026). Potential of small-molecule targeted drugs in combination with CAR-T cell therapy for hematologic lymphomas. Front. Immunol..

[283] Anurogo D., Luthfiana D., Anripa N., Fauziah A.I., Soleha M., Rahmah L., Ratnawati H., Wargasetia T.L., Pratiwi S.E., Siregar R.N. (2024). The art of bioimmunogenomics (BIGs) 5.0 in CAR-T cell therapy for lymphoma management. Adv. Pharm. Bull..

[284] Lai C., Hu J., Wu Z., He K., Li J., Zhu W., Liu C., He W., Xu K. (2026). A global multidimensional analysis of the chimeric antigen receptor T-cell therapy clinical trial landscape and development trends. Biomark. Res..

[285] Jin S., Liu Y., Zhang Y., Zhou F., You L., Zhang J. (2023). Bispecific T cell engagers targeting CD20/CD3 in B-cell lymphoma: Latest updates from 2023 EHA annual meeting. Ther. Adv. Chronic Dis..

[286] Sun L.L., Ellerman D., Mathieu M., Hristopoulos M., Chen X., Li Y., Yan X., Clark R., Reyes A., Stefanich E. (2015). Anti-CD20/CD3 T cell-dependent bispecific antibody for the treatment of B cell malignancies. Sci. Transl. Med..

[287] Dandamudi D.B., Konieczna I.M., Calabrese K.M., Wielgos-Bonvallet M., Kweekel C., Gresnigt-van den Heuvel E., Iyer R., Ellis P., Rodriguez L., Parikh A. (2025). Combinability of epcoritamab CD20-targeting T-cell engager and CD20 antibody-targeted therapies in B-cell non-Hodgkin lymphoma. Leuk. Lymphoma.

[288] Abou Dalle I., Dulery R., Moukalled N., Ricard L., Stocker N., El-Cheikh J., Mohty M., Bazarbachi A. (2024). Bi- and tri-specific antibodies in non-Hodgkin lymphoma: Current data and perspectives. Blood Cancer J..

[289] Labanca C., Martino E.A., Vigna E., Bruzzese A., Mendicino F., De Luca P., Lucia E., Olivito V., Fragliasso V., Neri A. (2024). Mosunetuzumab for the treatment of follicular lymphoma. Expert Opin. Biol. Ther..

[290] Sam J., Leclercq-Cohen G., Gebhardt S., Surowka M., Herter S., Lechner K., Relf J., Briner S., Varol A., Appelt B. (2025). Preclinical advances in glofitamab combinations: A new frontier for non-Hodgkin lymphoma. Blood.

[291] Zhang P., Zhang G., Wan X. (2023). Challenges and new technologies in adoptive cell therapy. J. Hematol. Oncol..

[292] Zhou L., Cheng W., Luo Q., Huang C., Yu L. (2025). Targeting epigenetic reprogramming in DLBCL and its interaction with the tumor microenvironment for novel therapeutic approaches. Int. Immunopharmacol..

[293] Fu L., Zhou X., Zhang X., Li X., Zhang F., Gu H., Wang X. (2025). Circulating tumor DNA in lymphoma: Technologies and applications. J. Hematol. Oncol..

[294] Loi S., Salgado R. (2025). AI-driven biomarkers for antibody-drug conjugates. Cancer Cell.

[295] Topol E.J. (2019). High-performance medicine: The convergence of human and artificial intelligence. Nat. Med..

[296] Esteva A., Robicquet A., Ramsundar B., Kuleshov V., DePristo M., Chou K., Cui C., Corrado G., Thrun S., Dean J. (2019). A guide to deep learning in healthcare. Nat. Med..

[297] Popescu D.C., Găman M.A. (2025). Artificial intelligence for risk stratification in diffuse large B-cell lymphoma: A systematic review of classification models and predictive performances. Med. Sci..

[298] Luan Y., Li X., Luan Y., Luo J., Dong Q., Ye S., Li Y., Li Y., Jia L., Yang J. (2024). Therapeutic challenges in peripheral T-cell lymphoma. Mol. Cancer.

[299] Claudel A., Cottereau A.-S., Bachy E., Itti E., Feugier P., Rossi C., Lemonnier F., Camus V., Daguindau N., Cartron G. (2025). Combined PET and ctDNA response as a predictor of POD24 for follicular lymphoma after first-line induction treatment. Blood.

[300] Leslie L.A., Skarbnik A.P., Bejot C., Stives S., Feldman T.A., Goy A.H. (2017). Targeting indolent non-Hodgkin lymphoma. Expert Rev. Hematol..

[301] Luke J.J., Patel M.R., Blumenschein G.R., Hamilton E., Chmielowski B., Ulahannan S.V., Connolly R.M., Santa-Maria C.A., Wang J., Bahadur S.W. (2023). The PD-1- and LAG-3-targeting bispecific molecule tebotelimab in solid tumors and hematologic cancers: A phase 1 trial. Nat. Med..

[302] Gribbin C., Chen J., Martin P., Ruan J. (2024). Novel treatment for mantle cell lymphoma—Impact of BTK inhibitors and beyond. Leuk. Lymphoma.

[303] Chu Y., Liu Y., Fang X., Jiang Y., Ding M., Ge X., Yuan D., Lu K., Li P., Li Y. (2023). The epidemiological patterns of non-Hodgkin lymphoma: Global estimates of disease burden, risk factors, and temporal trends. Front. Oncol..

[304] Al-Sawaf O., Jen M.-H., Hess L.M., Zhang J., Goebel B., Pagel J.M., Abhyankar S., Davids M.S., Eyre T.A. (2024). Pirtobrutinib versus venetoclax in covalent Bruton tyrosine kinase inhibitor-pretreated chronic lymphocytic leukemia: A matching-adjusted indirect comparison. Haematologica.

[305] Coombs C.C. (2023). Noncovalent BTK inhibitors in B-cell lymphoma. Clin. Adv. Hematol. Oncol..

[306] Caridà G., Martino E.A., Bruzzese A., Caracciolo D., Labanca C., Mendicino F., Lucia E., Olivito V., Rossi T., Neri A. (2025). Relapsed/refractory follicular lymphoma: Current advances and emerging perspectives. Eur. J. Haematol..

[307] Shirouchi Y., Maruyama D. (2026). Novel agents and therapeutic advances in T cell lymphoma. Int. J. Hematol..

[308] Akkad N., Huen A., Iyer S.P. (2026). Targeting cutaneous T-cell lymphoma in non-Hodgkin lymphoma: What’s new for investigational agents?. Expert Opin. Investig. Drugs.

[309] Woodcock J., LaVange L.M. (2017). Master protocols to study multiple therapies, multiple diseases, or both. N. Engl. J. Med..

[310] Dabrowska-Iwanicka A., Nowakowski G.S. (2024). DLBCL: Who is high risk and how should treatment be optimized?. Blood.

[311] Park J.J.H., Siden E., Zoratti M.J., Dron L., Harari O., Singer J., Lester R.T., Thorlund K., Mills E.J. (2019). Systematic review of basket trials, umbrella trials, and platform trials: A landscape analysis of master protocols. Trials.

[312] Gautam S., Gautam B., Shilpakar R., KC S., Kurmi O.P. (2024). CAR-T cell therapy in developing countries: How long should we wait?. J. Immunother. Cancer.

[313] Krown S.E. (2011). Cancer in resource-limited settings. J. Acquir. Immune Defic. Syndr..

[314] Shouse G. (2023). Update on bi-specific monoclonal antibodies for blood cancers. Curr. Opin. Oncol..

[315] Kubuschok B., Trepel M. (2017). Learning from the failures of drug discovery in B-cell non-Hodgkin lymphomas and perspectives for the future: Chronic lymphocytic leukemia and diffuse large B-cell lymphoma as two ends of a spectrum in drug development. Expert Opin. Drug Discov..

[316] Fanale M.A., Horwitz S.M., Forero-Torres A., Bartlett N.L., Advani R.H., Pro B., Chen R.W., Davies A., Illidge T., Huebner D. (2014). Brentuximab vedotin in the front-line treatment of patients with CD30+ peripheral T-cell lymphomas: Results of a phase I study. J. Clin. Oncol..

[317] Sawas A., Radeski D., O’Connor O.A. (2015). Belinostat in patients with refractory or relapsed peripheral T-cell lymphoma: A perspective review. Ther. Adv. Hematol..

[318] Eyre T.A., Cwynarski K., d’Amore F., de Leval L., Dreyling M., Eichenauer D.A., Ferreri A.J.M., Giné E., Kersten M.J., Ladetto M. (2025). Lymphomas: ESMO Clinical Practice Guideline for Diagnosis, Treatment and Follow-Up. Ann. Oncol..

[319] Tilly H., Morschhauser F., Sehn L.H., Friedberg J.W., Trněný M., Sharman J.P., Herbaux C., Burke J.M., Matasar M., Rai S. (2022). Polatuzumab Vedotin in Previously Untreated Diffuse Large B-Cell Lymphoma. N. Engl. J. Med..

[320] Coiffier B., Lepage E., Briere J., Herbrecht R., Tilly H., Bouabdallah R., Morel P., Van Den Neste E., Salles G., Gaulard P. (2002). CHOP Chemotherapy Plus Rituximab Compared with CHOP Alone in Elderly Patients with Diffuse Large-B-Cell Lymphoma. N. Engl. J. Med..

[321] Rummel M.J., Niederle N., Maschmeyer G., Banat G.A., von Grünhagen U., Losem C., Kofahl-Krause D., Heil G., Welslau M., Balser C. (2013). Bendamustine Plus Rituximab versus CHOP Plus Rituximab as First-Line Treatment for Patients with Indolent and Mantle-Cell Lymphomas: An Open-Label, Multicentre, Randomised, Phase 3 Non-Inferiority Trial. Lancet.

[322] Flinn I.W., van der Jagt R., Kahl B.S., Wood P., Hawkins T.E., Macdonald D., Hertzberg M., Kwan Y.-L., Simpson D., Craig M. (2014). Randomized Trial of Bendamustine-Rituximab or R-CHOP/R-CVP in First-Line Treatment of Indolent NHL or MCL: The BRIGHT Study. Blood.

[323] Salles G., Duell J., González Barca E., Tournilhac O., Jurczak W., Liberati A.M., Nagy Z., Obr A., Gaidano G., André M. (2020). Tafasitamab Plus Lenalidomide in Relapsed or Refractory Diffuse Large B-Cell Lymphoma (L-MIND): A Multicentre, Prospective, Single-Arm, Phase 2 Study. Lancet Oncol..

[324] Thieblemont C., Karimi Y.H., Ghesquieres H., Cheah C.Y., Clausen M.R., Cunningham D., Jurczak W., Do Y.R., Gasiorowski R., Lewis D.J. (2024). Epcoritamab in Relapsed/Refractory Large B-Cell Lymphoma: 2-Year Follow-Up from the Pivotal EPCORE NHL-1 Trial. Leukemia.

[325] Dickinson M.J., Carlo-Stella C., Morschhauser F., Bachy E., Corradini P., Iacoboni G., Khan C., Wróbel T., Offner F., Trněný M. (2022). Glofitamab for Relapsed or Refractory Diffuse Large B-Cell Lymphoma. N. Engl. J. Med..

[326] Budde L.E., Sehn L.H., Matasar M., Schuster S.J., Assouline S., Giri P., Kuruvilla J., Canales M., Dietrich S., Fay K. (2022). Safety and Efficacy of Mosunetuzumab, a Bispecific Antibody, in Patients with Relapsed or Refractory Follicular Lymphoma: A Single-Arm, Multicentre, Phase 2 Study. Lancet Oncol..

[327] Locke F.L., Ghobadi A., Jacobson C.A., Miklos D.B., Lekakis L.J., Oluwole O.O., Lin Y., Braunschweig I., Hill B.T., Timmerman J.M. (2019). Long-Term Safety and Activity of Axicabtagene Ciloleucel in Refractory Large B-Cell Lymphoma (ZUMA-1): A Single-Arm, Multicentre, Phase 1–2 Trial. Lancet Oncol..

[328] Abramson J.S., Palomba M.L., Gordon L.I., Lunning M.A., Wang M., Arnason J., Mehta A., Purev E., Maloney D.G., Andreadis C. (2020). Lisocabtagene Maraleucel for Patients with Relapsed or Refractory Large B-Cell Lymphomas (TRANSCEND NHL 001): A Multicentre Seamless Design Study. Lancet.

